# Evidence of microbiome contribution to the escalation of pyrethroid resistance in the major malaria vectors *Anopheles gambiae* s.s. and *Anopheles funestus* s.s

**DOI:** 10.1186/s12866-025-04114-0

**Published:** 2025-07-02

**Authors:** Fleuriane Metissa Djondji Kamga, Leon M. Jean Mugenzi, Vanessa Brigitte Ngannang-Fezeu, François Sougal Ngambia Freitas, Calmes Ursain Bouaka Tsakeng, Maurice Marcel Sandeu, Magellan Tchouakui, Charles Sinclair Wondji

**Affiliations:** 1 Centre for Research in Infectious Diseases (CRID), P.O. Box 13591, Yaoundé, Cameroon; 2 Department of Microbiology, Faculty of Science, University of Yaoundé I, P.O. Box 812, Yaoundé, Cameroon; 3 Department of Microbiology and Infectious Diseases, School of Veterinary Medicine and Sciences, University of Ngaoundéré, P.O. Box 454, Ngaoundéré, Cameroon; 4 Vector Biology Department, Liverpool School of Tropical Medicine, Liverpool, L3 5QA UK

**Keywords:** Malaria, Microbiome, Pyrethroids, Resistance escalation, *An. funestus* s.s., *An. gambiae* s.s

## Abstract

**Background:**

Exacerbation of pyrethroid resistance severely jeopardises the effectiveness of malaria vector control efforts. However, the mechanisms enabling the vectors to now survive exposure to very high doses of pyrethroids remain unclear. Here, using High-throughput sequencing of the 16 S ribosomal RNA gene coupled with antibiotic treatment, we provide evidence linking the mosquito microbiome to the escalation of pyrethroid resistance in major African malaria vectors, *Anopheles gambiae* (s.s.) and *Anopheles funestus* (s.s.).

**Results:**

Phenotypic characterisation of *An. gambiae* (s.s.) and *An. funestus* (s.s.) populations revealed a high level of resistance to pyrethroid in both species, with mortality rates < 91% at 10x the diagnostic dose of each insecticide. A significant difference in bacterial composition was observed in *An. gambiae* s.s. between resistant mosquitoes exposed to 1X and 10X the diagnostic dose of permethrin, and the susceptible strains (PERMANOVA-F: 8.06; *p* = 0.02). The abundance of *Pseudomonas_1* (Log2FC: 4.42, *p* = 0.0001) and *Burkholderia_1* (Log2FC: 4.95, *p* = 0.001) bacteria were consistently associated with mosquitoes surviving 1X and 10X the diagnostic concentrations of permethrin, respectively, while *Serratia_2* bacteria was mostly associated with insecticide susceptibility. In the *An. funestus* s.s. strain, there was no significant difference in bacterial alpha- and beta-diversity between the FUMOZ-R (exhibiting normal deltamethrin resistance) and FUMOZ-HR (selected for high deltamethrin resistance), suggesting a minimal impact of selection pressure on bacterial composition. However, in FUMOZ-HR, there was an increase in the abundance of *Rahnella* (Log2FC: 15.954, *p* = 9.73 E-12) and *Leucobacter* (Log2FC: 7.6, *p* = 0.008) bacteria, indicating their potential role in worsening deltamethrin resistance. Furthermore, treating resistant mosquitoes (both *Anopheles* species) with broad-spectrum bactericidal antibiotics (penicillin/streptomycin) via sugar solution increased their susceptibility to various diagnostic doses of permethrin and deltamethrin in WHO pyrethroid intensity bioassays.

**Conclusion:**

Overall, our study emphasises the potential role of the microbiome in the escalation of insecticide resistance in *Anopheles* mosquitoes, identifying key bacterial strains associated with insecticide resistance and susceptibility. These candidate bacteria warrant further investigation to elucidate the mechanisms by which they contribute to the escalation of pyrethroid resistance.

**Supplementary Information:**

The online version contains supplementary material available at 10.1186/s12866-025-04114-0.

## Background

Malaria is the most devastating vector-borne disease, with over 263 million cases reported in 2023, marking a significant increase from previous years [1]. The disease resulted in approximately 597,000 deaths annually, primarily affecting children under five years and pregnant women in sub-Saharan Africa. Insecticide-based interventions, such as long-lasting insecticidal nets (LLINs) and indoor residual spraying (IRS), have been pivotal in reducing malaria incidence, averting about 68% of cases since the early 2000s [2]. However, the emergence of insecticide resistance among malaria vectors poses a significant threat to these control strategies. Resistance to pyrethroids, which are widely used in bednets, the primary vector control tool, has escalated alarmingly [3, 4]. For instance, studies revealed that *Anopheles funestus* populations across Africa are now resistant to higher doses up to ten times (10X) the diagnostic dose of pyrethroids, reducing the efficacy of pyrethroid-impregnated nets in West Africa [5], East Africa [4], Southern Africa [6] and Central Africa [7, 8]. Similarly, *Anopheles gambiae* s.l. has exhibited high levels of resistance to all insecticides used for vector control, especially pyrethroids even at 10X diagnostic concentration in Uganda [4], Ghana [5, 9], Cote d’Ivoire [10], Nigeria [11], Democratic Republic of Congo [8, 12] and Cameroon [13, 14].

Mosquito vectors can develop resistance to insecticides through several mechanisms, including metabolic enzymatic detoxification, target site modification, reduced insecticide penetration, and behavioural avoidance [15–20]. Recent studies have also highlighted the insect microbiome as a significant driver of insecticide resistance [21, 22]. The mosquito microbiota is primarily composed of Gram-negative bacteria [23] that can be acquired from breeding habitats [24, 25] or transmitted through transovarial means [26]. Some of these symbionts can enhance the host’s immunity and biochemical detoxification processes [24, 27]. Emerging evidence indicates that specific gut bacteria may actively degrade insecticides or modify the host’s physiological responses. This relationship highlights the complexity of interactions between mosquito microbiota and their survival following insecticide exposure. In some hosts, the association is directly linked to insecticide degradation by specific microbes within the gut. This has particularly been shown with the symbiont *Burkholderia*, capable of breaking down fenitrothion (an organophosphate) in the agricultural pest *Riptortus pedestris* (The bean bug) [28]. Similarly, the symbiont *Serratia oryzea* has been shown to metabolise deltamethrin in its mosquito host *Aedes albopictus* [29]. In addition, studies suggest that the bacteria-host association is mediated by physiological interactions that indirectly influence the expression of the host’s endogenous detoxification genes and resistance phenotype. For instance, treating the temephos-resistant strain of *An. stephensi* with tetracycline, reduced the activity of three enzyme families (esterases, glutathione S-transferases, and acetylcholinesterase) while reinstating the mosquito’s susceptibility to this insecticide [30]. A similar observation has been reported in *An. arabiensis*, where a decrease in resistance to deltamethrin (a pyrethroid) and malathion (an organophosphate) was observed after treating mosquitoes with antibiotics [31]. Despite this evidence, it remains unknown if the microbiota plays a similar role in the intensification of pyrethroid resistance that has been observed across Africa. Understanding how the microbiome contributes to the exacerbation of resistance levels is crucial for developing alternative and effective malaria control strategies. There is also a crucial need to understand if these microbial contributions can induce cross-resistance between pyrethroids and other insecticides, which could help improve the design of more resilient next-generation insecticides. Our study investigated the contribution of mosquito microbiota to pyrethroid resistance escalation in the two major malaria vectors, *An. funestus* s.s. and *An. gambiae* s.s. We reveal that specific bacterial strains are associated with the ability of mosquitoes to survive higher doses of pyrethroids. This was further demonstrated when increased susceptibility to insecticides was observed in mosquitoes treated with antibiotics.

## Materials and methods

### Mosquito collection, rearing, and processing

From September to November 2022, mosquitoes were collected in Nkolondom village, located in Yaoundé (3°57′18″ N, 11°29′36″ E) (Additional Fig. 1), an area known for harbouring *An. gambiae* species with high levels of pyrethroid resistance [13, 32]. The Kisumu susceptible reference strain of *An. gambiae* s.s. was used as a control. Larvae and pupae were collected using the dipping method [33] and reared under controlled conditions (25 °C (± 2 °C), and 70% (± 20%), with a 12 h/12 h day/night photoperiod) until adult emergence at the Center for Research in Infectious Diseases (CRID) insectary.

Two strains of *An. funestus* s.s. were also used: FUMOZ-R, and FUMOZ-HR. The FUMOZ-R strain, derived from southern Mozambique, exhibits a typical resistance profile to pyrethroids [34] and has been maintained as a colony at CRID. The FUMOZ-HR strain underwent selection over four generations using a 10X diagnostic concentration of deltamethrin, resulting in highly deltamethrin-resistant mosquitoes. These strains were reared in the CRID’s insectary under the same controlled conditions, fed with TetraMin™ baby fish in mineral water [35].

### Insecticide susceptibility profiling

The resistance intensity profile was assessed using the WHO tube test protocol [36]. Non-blood-fed adult females, 4–5 days old, were exposed for 1 h to a discriminating concentration (DC) of pyrethroid at 1X, 5X, and 10X. For each test, 100 females were divided into four replicates of 20–25 mosquitoes, which were aspirated and transferred to tubes containing insecticide-treated papers. In parallel, 40–50 females were used as controls in non-impregnated tubes without insecticide. Bioassays were performed under specific conditions: temperature 25 °C (± 2 °C), and 70% (± 20%) relative humidity. Mortality rates were recorded 24 h after exposure. According to WHO guidelines, resistance and intensity resistance status were assigned [36]. Dead mosquitoes were conserved with silica gel, while survivors were stored in RNA later and kept at −80 °C, for future genomic analyses.

### DNA extraction

Pyrethroid-exposed and Unexposed *Anopheles* mosquitoes were used to investigate the relationship between the presence of specific bacterial taxa and the escalation of insecticide resistance. To avoid microbial contamination, the surface of each mosquito’s cuticle was sterilised twice with 70% ethanol (v/v) and rinsed with sterile 1X PBS (Phosphate Buffer Solution) buffer [37]. Briefly, 200 µl of 70% ethanol was added to a reaction tube containing individual mosquitoes, which were vortexed for approximately 30 s, followed by a gentle rinse with 200 µl of sterile 1X PBS solution. Genomic DNA was isolated and purified from the whole mosquito using the GeneJET Genomic DNA extraction kit (Thermo Scientific™, #K0722) according to the manufacturer’s instructions. During the DNA extraction process, three blank controls of 1X PBS solution (Rinse mosquito), and three other blanks of 70% ethanol (for sterilising mosquitoes and instruments) were included to assess cross-contamination. According to the different resistance phenotypes (*An. gambiae* s.s.: *Unexposed*,* Survivors_1X*,* Survivors_10X*,* Kisumu (susceptible); An. funestus* s.s.: FUMOZ-R, and FUMOZ-HR), DNA of nine individuals were pooled per replicate, resulting in a total of 27 mosquitoes/phenotype (Additional Table S.1). The same pooling procedure was applied to the blank controls. The final concentration of each pooled DNA was adjusted to 5 ng/µl for normalisation.

### 16S rRNA amplification and library preparation

The different phenotypes, along with blank controls, were sequenced on the Illumina Miseq system platform at the Genomic lab of CRID, using the 16 S Metagenomic Sequencing Library Preparation protocol (Illumina TM, San Diego, CA, USA) [38]. The negative water control underwent the same processing as the other samples. For the amplification of the 16 S rRNA gene, two specific primers targeting the v3-v4 region (approximately 550 bp) were used:

- v3-F 5’-TCGTCGGCAGCGTCAGATGTGTATA AGAGACAGCCTACGGGNGGCWGCAG-3’;
- v4-R 5’-GTCTCGTGGGCTCGGAGATGTGTAT AAGAGACAGGACTACHVGGGTAT CTAATCC-3’.

The amplification reaction was conducted in a total volume of 25 µL, which included 12.5 µL of 2X KAPA HiFi HotStart Ready (KAPA Biosystems), 2.5 µL of pooled mosquito DNA, and 5 µL of each primer (1 µM). The amplification was performed on a T100 Thermal Cycler (Bio-Rad) using the following cycling conditions: initial denaturation at 95 °C for 3 min; followed by 25 cycles of 95 °C for 30 s, 55 °C for 30 s, and 72 °C for 30 s, concluding with a final extension at 72 °C for 5 min. The PCR amplicons (approximately 550 bp) were purified using Agencourt AMPure XP beads (Beckman Coulter Inc., USA). Subsequently, 5 µL of these purified PCR products were used in a second round of PCR for library preparation, where dual indexes (i5 and i7) and adapters were attached using the MiSeq reagent v3 kit (Paired-end 2 × 300 bp) (Illumina catalogue, CA, USA). Another clean-up step was performed using AMPure XP beads, and the quality of the generated libraries was confirmed using a bioanalyzer (TapeStation 4200) (The DNA Integrity Number (DIN) value: ≥ 7, Expected size ~ 630 bp). The resulting sequencing libraries were quantified with a Qubit^®^ 3.0 Fluorometer (Thermo Fisher Scientific, USA), normalised and pooled to a concentration of 4 nM. Finally, the pooled libraries and the 5% PhiX solution (serving as an internal control for sequencing) were denatured according to Illumina guidelines and loaded onto the Illumina MiSeq flow cell for cluster generation. Sequencing was performed on the Illumina MiSeq using MiSeq 2 × 300 cycle paired-end sequencing kits at the Genomic lab at CRID.

### Data processing

The generated raw paired-end sequencing reads were demultiplexed and analysed using the Mothur v.1.44.3 software [39], following a modified pipeline [40]. The pipeline for the analyses of the 16 S rRNA dataset is freely available at: https://github.com/Djondji/Metagenomics-16S. Sequences were merged to create contiguous sequences for each pool, and primers/adapters were trimmed. Quality filtering was then performed to remove ambiguous bases, ensuring the integrity of the dataset. The obtained dataset was reduced to single sequences and aligned to the 16 S rRNA v3-v4 region of the SILVA.v.123 reference database. Overhangs at both ends were removed, along with unique sequences whose abundance was less than 0.01%, likely due to sequencing errors. A supplementary filtering step was performed to eliminate chimeric, eukaryotic/mitochondrial, and chloroplastic sequences. A distance matrix was created from the clean sequences, which were then classified into operational taxonomic units (OTUs). Using the count-file, an OTU table containing the sample ID, all OTUs, and their abundances was generated. This OTU table was imported into R software and the *MicrobiomeAnalyst* platform [41] for microbial community analysis, including bacterial characterisation, diversity assessments, differential abundance estimation, statistical analysis, and data visualisation. A rarefaction curve was plotted to evaluate whether the sequencing depth was sufficient to accurately represent all the taxa present in individual mosquito pools.

### Bacterial diversities

Alpha and beta diversity metrics were compared among samples with different resistance phenotypes using the R software (code available at: https://github.com/Djondji/Metagenomics-16S). The Chao1 and Shannon alpha-diversity indexes were estimated to assess the bacterial richness or evenness. Statistical comparisons between groups were conducted using the non-parametric Kruskal-Wallis test, followed by pairwise comparisons using the Wilcoxon test. Dissimilarity in bacterial composition across samples was evaluated using the Bray-Curtis index and visualised through Principal Co-ordinates Analysis plots (PCoA). The differences between groups were further analysed using Permutational Multivariate Analysis of Variance (PERMANOVA 999), with a significance threshold set at a *p*-value of < 0.05. These analyses provided insights into the diversity and composition of the bacterial communities associated with different resistance phenotypes.

### Differential abundance testing

The differential abundance analysis was performed using the DESeq 2 method [42, 43] via the *MicrobiomeAnalyst* platform (https://www.microbiomeanalyst.ca/). This approach employs a negative binomial distribution to detect variations in bacterial read counts between groups. Its normalisation considers the differences between library sizes and compositions. The resulting statistic, Log two-fold change, and its *p*-value cutoff (< 0.05) were used to identify differentially abundant features between different groups.

### Antibiotic treatment of field *An. gambiae* s.s. mosquitoes and *An. funestus*s.s. strains

To validate the role of the bacterial community in exacerbating pyrethroid resistance, female mosquitoes from both species were provided daily with a sterile 10% sucrose solution supplemented with 100 U/mL penicillin and 100 µg/mL streptomycin (Pen/Strep) according to the methodology proposed by Ramirez et al. [44]. This combination of antibiotics provides a broad-spectrum synergistic antimicrobial action, effectively suppressing both Gram-positive (Penicillin inhibits the cross-linking of peptidoglycan within the bacteria cell wall) and Gram-negative bacteria (Streptomycin inhibits protein synthesis of the 30 S ribosomal RNA subunit). Mosquitoes receiving the antibiotic mixture (Pen/Strep) were classified as the treated group, while those maintained on a 10% sugar solution only, were designated as the untreated group. The mosquitoes were reared under controlled conditions in a Memmert IN/IF 30 incubator at 25 °C, with 80% humidity, and a light/dark cycle of 12:12 h. The antibiotic treatment was administered for five days before insecticide exposure, and all sugar solutions were replaced every 24 h to ensure a continuous supply of nutrients.

### Efficacy of the antibiotic treatment and its effect on mosquitoes’ survivorship

The effectiveness of the P/S antibiotic treatment was evaluated by comparing the relative abundance of the *Asaia* symbiont (well-established and characterised bacteria in malaria vectors, notably at CRID [45]) between the treated and untreated groups using quantitative PCR (qPCR) as described by Jeffries et al. (2018) [46]. The AsaH1_F and Asar_R primers were employed to estimate the bacterial load of *Asaia* [45], relative to the ribosomal protein S7 housekeeping gene of *An. gambiae* (VectorBase ID: AGAP010592) and *An. funestus* (VectorBase ID: AFUN007153) as detailed in additional Table S.2. Each qPCR reaction was performed in duplicate in a volume containing: 2 µl of genomic DNA, 5 µl of SYBR Green PCR Master Mix 2X (Applied Biosystems™), 1 µM of each primer, and 1 µL of nuclease-free water. Amplification conditions were: 95 °C for 15 min; denaturation at 95 °C for 10 s followed by 40 cycles at 95 °C for 10 s; 65.5 °C for 1 min; and 97 °C for 1s. The differences in the relative abundance of *Asaia* spp. between the treated and untreated groups was assessed using the Mann-Whitney non-parametric test, conducted with GraphPad Prism 8.2.0.

To evaluate the effect of antibiotic treatment on mosquito survivorship, multiple experimental cohorts of antibiotic treatment were established, and the mortality rates of mosquitoes were reported daily for 5 days post-treatment. Survival curves for the treated and untreated groups were plotted using GraphPad Prism 8.2.0. A Kaplan-Meier estimator was utilised, and a log-rank test was applied to determine the significance of differences in survival between the two groups (Treated and Untreated).

### Impact of antibiotic treatment on the expression of key P450 metabolic resistance genes

Quantitative reverse-transcription PCR (qRT-PCR) was conducted to assess whether the antibiotic treatment modulated the expression of key metabolic resistance genes in mosquitoes, specifically focusing on most over-expressed P450s in FUMOZ-R (*CYP6P9a*,* CYP6P9b*) [47] and *An. gambiae* s.s. (*CYP9K1*) [13]. Total RNA was extracted from three biological replicates (each consisting of ten mosquitoes) from the treated, untreated, FANG and Kisumu strains on day 5 post-treatment. The extraction was performed using the Picopure RNA isolation kit (Life Technologies, Carlsbad, CA, USA). Following RNA extraction, complementary DNA (cDNA) was synthesized using Superscript III (Invitrogen), with oligo-dT20 primers, and RNase H, adhering to the manufacturer’s instructions. qRT-PCR was conducted on an MX3005 real-time PCR system (Agilent) in a total volume of 20 µl comprising 10 µl of Brilliant III Ultra-Fast SYBR Green qPCR Master Mix (Agilent), 0.6 µM of primers, 7.8 µl of sterile water and 1 µl of cDNA. The reaction was carried out according to a specific amplification program previously described [48]. The relative expression levels of each gene were calculated using the 2^−ΔΔCT^ method [49], normalized with the housekeeping genes of *An. funestus* s.s. (ribosomal protein S7 (RSP7; AFUN007153) and actin 5 C (AFUN006819)) and *An. gambiae* s.s. (Elongation factor (AGAP000883) and Ribosomal Protein S7 (AGAP010592)). The list of all the primers used for the experiment is summarized in additional Table S.2. The fold changes in gene expression between the treated and untreated groups were statistically compared using the Welch corrected test.

### Impact of antibiotic treatment on susceptibility profile

After the 5 days of antibiotic treatment, both the treated and untreated mosquitoes were exposed to increasing concentrations of permethrin and deltamethrin (1X, 5X, and 10X) as described in the WHO protocol [36]. Mosquito mortality rates were calculated along with the standard error of the mean (SEM). A Student’s t-test was performed to compare mortality rates between the two groups to assess the effect of the antibiotic on the resistance profile.

## Results

### Insecticide resistance profile of the mosquito population tested

**Fig. 1:**
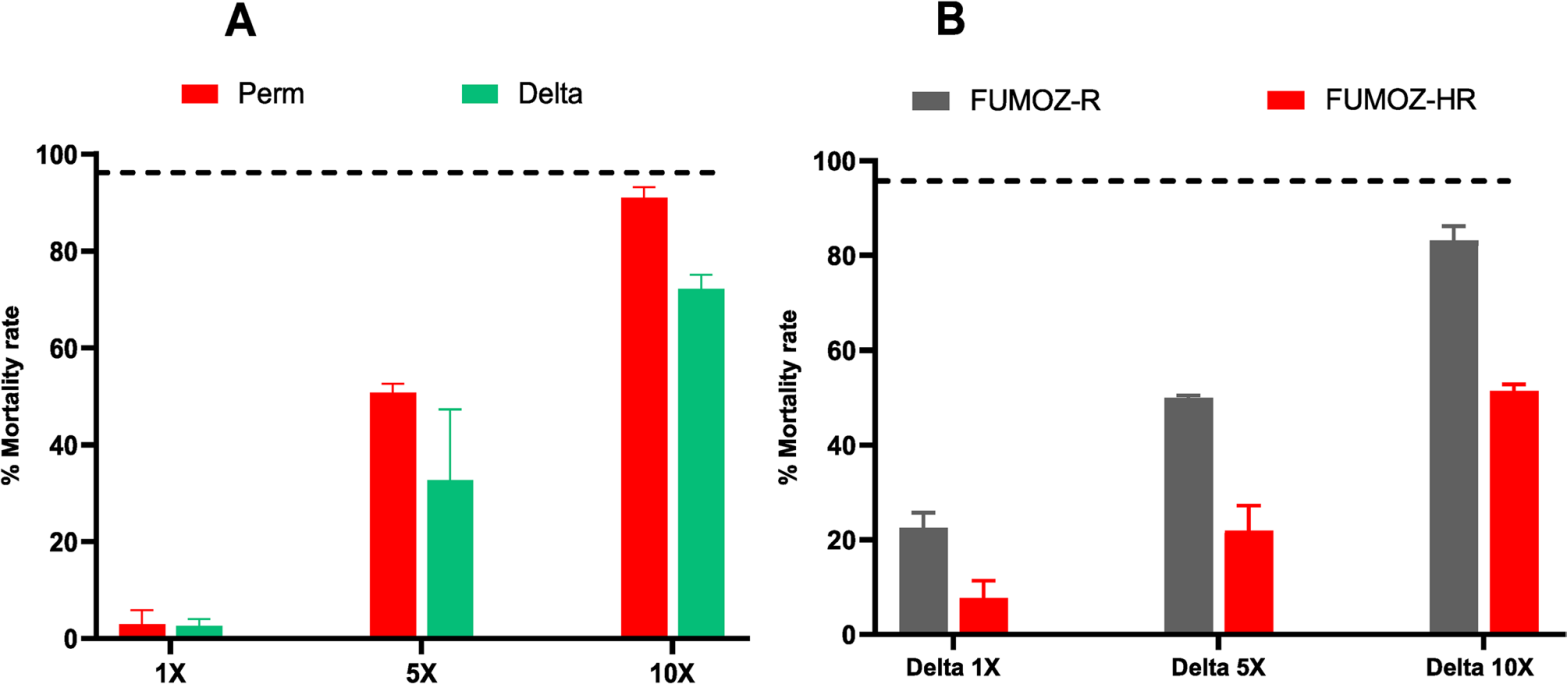
Insecticide susceptibility profile of *Anopheles* mosquitoes: **A** Resistance profile of *An. gambiae* field mosquitoes from Nkolondom. **B** Susceptible profile of FUMOZ laboratory strains, including selected (FUMOZ-HR) and unselected (FUMOZ-R). All mosquitoes were exposed to 1X, 5X, and 10X diagnostic doses of pyrethroids, with data reported as mean mortalities ± SEM (Standard error of mortality rates). Insecticides tested: Permethrin (Perm), and Deltamethrin (Delta)

An intense resistance profile to pyrethroids was evident in *An. gambiae* s.s. from Nkolondom at all the concentrations of insecticide tested. The observed mortality rates at the diagnostic 1X were notably low for both type I [permethrin (2.94 ± 2.94%)] and type II [deltamethrin (2.66 ± 1.37%)] pyrethroids. This pattern of low mortality was consistent even at higher concentrations (5X and 10X), confirming a high resistance level (see Fig. 1A). This situation highlights the severe challenge posed by pyrethroid resistance in the peri-urban areas of Yaoundé, which is likely attributed to the extensive and improper use of pesticides in local agricultural practices [13, 50]. Regarding the selection process for the FUMOZ strain, a marked increase in resistance intensity to deltamethrin was observed in the FUMOZ-HR strain. The mortality rate in this strain was significantly lower, with only about 52% at 10X diagnostic dose, compared to 82% for the unselected line (see Fig. 1B).

### 16S rRNA sequencing quality and filtering

The v3-v4 region of the 16S rRNA gene was studied in 162 female mosquitoes, divided into 18 pools of nine mosquitoes each. From this analysis, a total of 12,661,699 paired-end reads were generated. After processing and filtering for quality, 3,684,835 cleaned sequences remained, with the number of reads per sample ranging from 65,129 to 474,399. The rarefaction curves of each sample reached a plateau from a library size of approximately 6000 reads (Additional Fig. 2), achieving 99% coverage (Additional Table S.2). This indicates that the sequencing effort was sufficient to capture most or even all the bacterial taxa present in the samples as previously shown in similar studies [43, 51].

### Bacterial characterisation

**Fig. 2:**
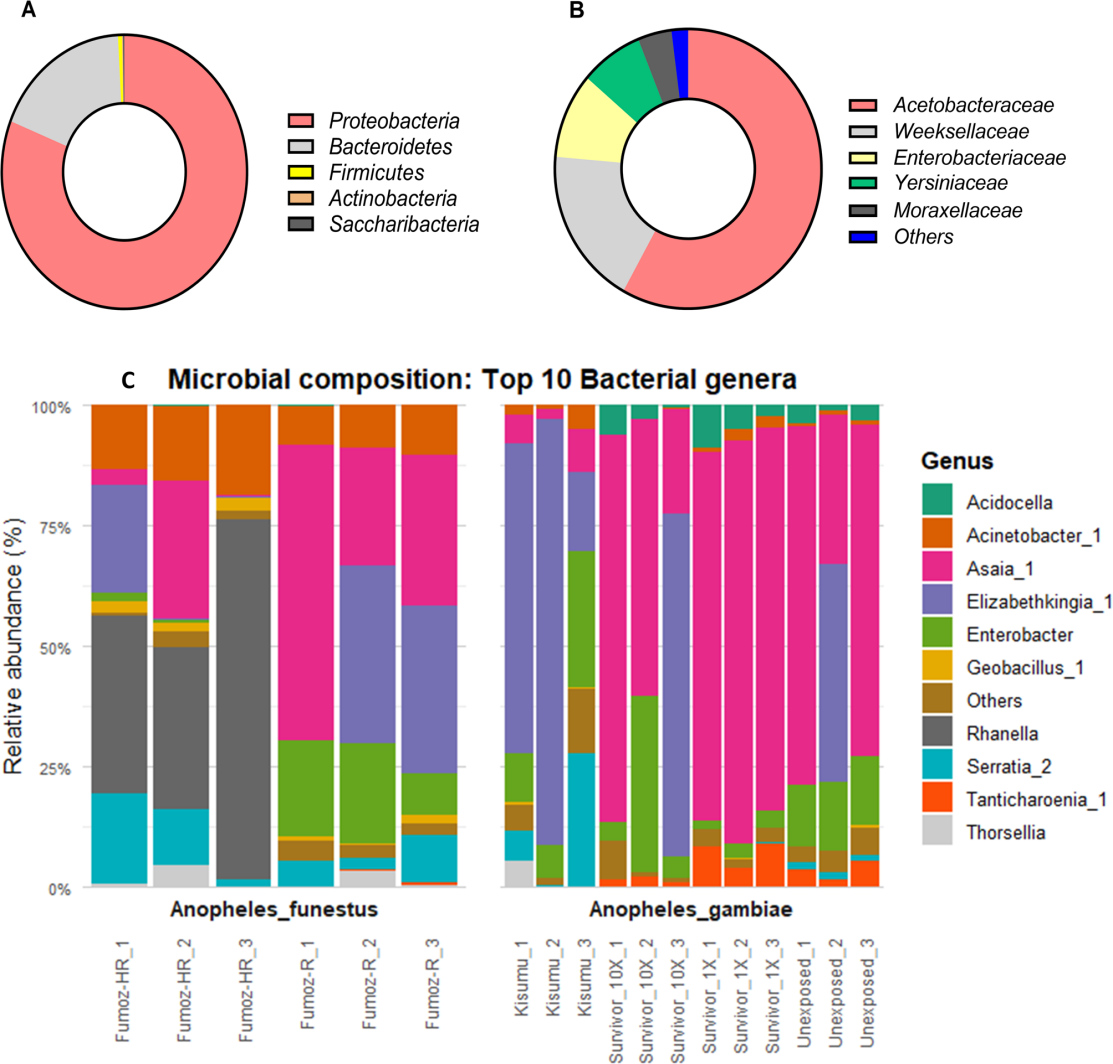
Bacterial characterisation in *Anopheles* mosquitoes includes: **A** Proportion of phyla in overall samples. **B** Proportions of bacterial families in overall samples. **C** Relative abundance of the top 10 bacteria genera identified across samples

The distribution of bacterial taxa across the 18 pools (162 mosquitoes) at the phylum, family, and genus levels is detailed in Fig. 2. One hundred eighty operational taxonomic units (OTUs) were identified in the overall samples examined. These OTUs belonged to 6 phyla, 33 families, and 59 genera. The most abundant phyla were *Proteobacteria* and *Bacteroidetes* with 81.19% and 18.15% relative abundance (Fig. 2A). We found five dominant families in the core microbiome: *Acetobacteraceae* (57.56%), *Weeksellaceae* (18.13%), *Enterobacteriaceae* (9.82%), *Yersiniaceae* (7.53%), and *Moraxellaceae* (4.06%) (Fig. 2B). At the genus level, the predominant bacterial genus was *Asaia_1*, one of the major symbionts of *Anopheles* mosquitoes, with a proportion of 52.04%. The other abundant genera were *Elizabethkingia_1* (17.84%), *Enterobacter* (9.26%), *Rahnella* (4.51%), *Acinetobacter_1* (3.95%), *Serratia_2* (3.01%) *Acidocella* (2.81%), and *Tanticharoenia_1* (3.64%). The remaining 3.87% was shared between other bacterial genera less represented and unclassified genera (Fig. 2C).

In *An. gambiae* s.s. mosquitoes, the dominant phyla in the susceptible population (*Kisumu* strain) were *Bacteroidetes* and *Proteobacteria*, while most of the sequences from field mosquitoes (*Unexposed*, *Survivor_1X*, and *Survivors_10X*) were dominated by *Proteobacteria* and *Bacteriodetes* (Table 1). The same pattern was observed at the family and genus levels. *Weeksellaceae* and *Enterobacteriaceae* were the most abundant families in the *Kisumu* strain, while *Acetobacteriaceae* and *Enterobacteriaceae* have a higher proportion in the field population. *Elizabethkingia_1* was by far the most represented genus in the susceptible group, while *Asaia_1* dominated the field mosquitoes.

In the *An. funestus* s.s. strain, there was a higher proportion of *Yersiniacaea* and *Moraxellacaea* families in the FUMOZ-HR. *Rahnella* and *Acinetobacter* genera were more abundant in FUMOZ-HR compared to FUMOZ-R. On the other hand, OTUs annotated as *Asaia_1* and *Elizabethkingia_1* were predominant in the FUMOZ-R strain. For a detailed overview of the microbiota composition and the frequencies of the predominant phyla, families, and genera, refer to Table 1.

**Table 1:** Frequencies (with ranges) of the dominant phyla, families, and genera according to the resistance status of *Anopheles* mosquitoes

Mosquitoes species	Phenotypes	Classification	Bacteria	% Mean proportion (Ranges)
*An. gambiae* s.s.	Kisumu (*N* = 27)	Phyla	*Bacteroidetes*	56.39 (16.38–88.33)
			*Proteobacteria*	43.08 (11.42–83.52)
		Family	*Weeksellaceae*	56.35 (16.38–88.31)
			*Enterobacteriaceae*	16.85 (6.83–28.38)
		Genus	*Elizabethkingia_1*	56.33 (16.37–88.30)
			*Enterobacter*	15.04 (6.83–28.32)
			*Serratia_2*	11.50 (0.40–27.93)
	*Unexposed* (*N* = 27)	Phyla	*Proteobacteria*	82.71 (53.87–99.27)
			*Bacteroidetes*	16.79 (0.23–45.91)
		Family	*Acetobacteraceae*	64.17 (33.71–81.51)
			*Enterobacteriaceae*	16.76 (0.22–45.83)
		Genus	*Asaia_1*	57.85 (30.73–74.17)
			*Elizabethkingia_1*	15.20 (0.06–45.45)
			*Enterobacter*	13.69 (12.75–14.21)
	*Survivors_1X* (*N* = 27)	Phyla	*Proteobacteria*	99.70 (99.66–99.73)
			*Bacteroidetes*	0.1 (0.06–0.12)
		Family	*Acetobacteraceae*	92.32 (90.87–93.77)
			*Enterobacteriaceae*	2.77 (1.52–3.64)
		Genus	*Asaia_1*	79.76 (76.49–83.41)
			*Tanticharoenia_1*	7.19 (3.99–9.17)
			*Acidocella*	5.37 (2.32–8.88)
	*Survivors_10X* (*N* = 27)	Phyla	*Proteobacteria*	74.47 (28.30–99.76)
			*Bacteroidetes*	23.86 (0.05–71.36)
		Family	*Acetobacteraceae*	57.82 (23.08–87.99)
			*Weeksellaceae*	23.86 (0.05–71.36)
		Genus	*Asaia_1*	53.15 (21.71–80.46)
			*Elizabethkingia_1*	23.82 (0.03–71.30)
			*Enterobacter*	14.91 (3.86–36.72)
*An. funestus* s.s.	FUMOZ-HR (*N* = 27)	Phyla	*Proteobacteria*	89.29 (74.97–97.43)
			*Bacteroidetes*	7.83 (0.27–22.48)
		Family	*Yersiniacaea*	59.04 (45.31 76.23)
			*Moraxellacaea*	16.07 (13.69–18.62)
		Genus	*Rahnella*	48.35 (33.6–74.7)
			*Acinetobacter_1*	15.91 (13.4–18.6)
			*Serratia_2*	10.69 (1.5–18.9)
	FUMOZ-R (*N* = 27)	Phyla	*Proteobacteria*	74.52 (62.62–97.81)
			*Bacteroidetes*	24.24 (1.05–36.85)
		Family	*Acetobacteraceae*	39.38 (24.66–61.49)
			*Weeksellaceae*	24.20 (1.05–36.77)
		Genus	*Asaia_1*	39.02 (24.4–61.4)
			*Elizabethkingia_1*	23.85 (0.06–36.75)
			*Enterobacter*	16.32 (8.48–20.78)

N: Number of mosquitoes tested per phenotype

### Bacterial diversities according to the resistance status of *Anopheles* mosquitoes

**Fig. 3:**
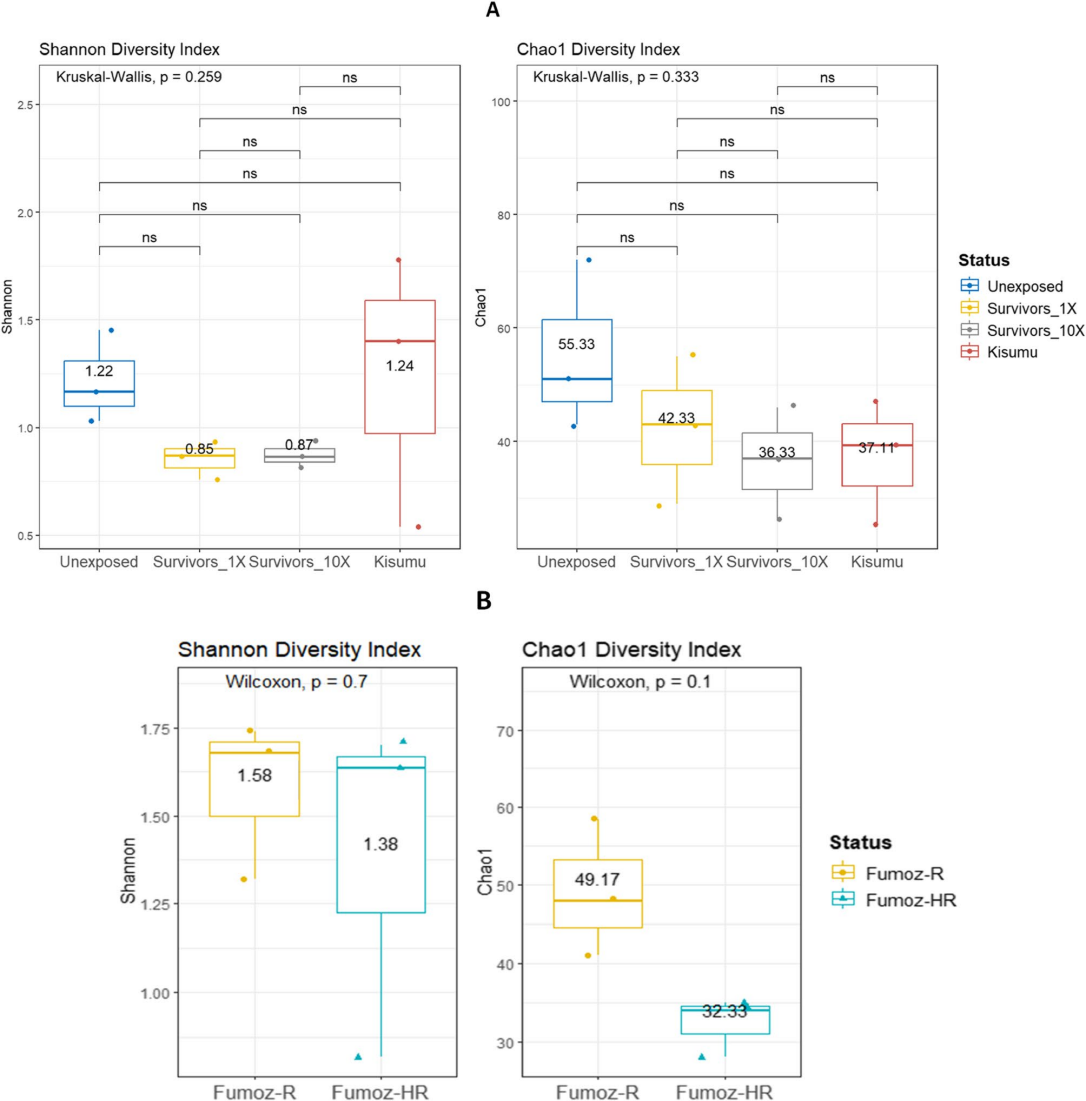
Microbiome alpha-diversity based on the resistance phenotypes of: **A** *An. gambiae* (s.s.) field mosquitoes and (**B**) *An. funestus* (s.s.) lab strains mosquitoes

Alpha-diversity analysis in *An. gambiae* s.s. revealed no statistically significant difference in bacterial richness and evenness across mosquito resistance phenotype groups (Shannon index [Kruskal-Wallis test], *p* = 0.26; Chao1 index [Kruskal-Wallis test], *p* = 0.33) (Fig. 3A). A similar outcome was obtained when comparing alpha diversity across phenotypes in *An. funestus* s.s. (Shannon index [Wilcoxon test], *p* = 0.7; Chao1 index [Wilcoxon test], *p* = 0.1) (Fig. 3B).

**Fig. 4:**
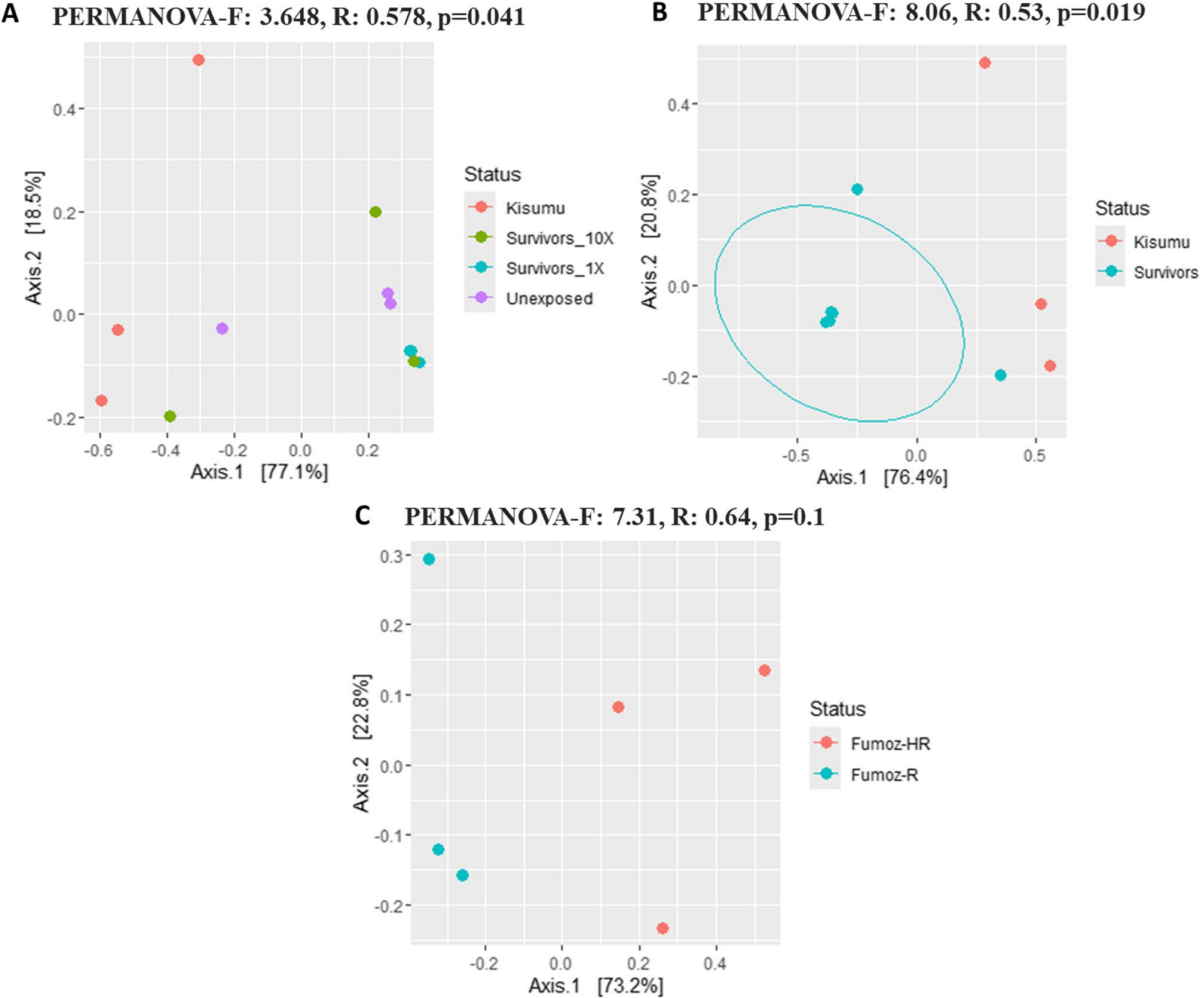
Representation of beta-diversity using principal coordinates analysis (PCoA) on the Bray-Curtis dissimilarity index. **A** Visualisation of patterns and variations in beta-diversity across different resistance phenotypes of *An. gambiae* s.s. **B** Comparison of bacterial beta-diversity between susceptible (*Kisumu*) and *Survivors* (1X and 10X) of *An. gambiae* s.s. mosquitoes. **C** Assessment of bacterial composition between resistance phenotypes of *An. funestus* s.s. mosquitoes

Beta-diversity analysis using the principal coordinates analysis (PCoA) with the Bray-Curtis dissimilarity index revealed distinct clusters in the microbiota of field *An. gambiae* s.s., separate from the susceptible Kisumu strain, except for two field samples that clustered closely with Kisumu. Despite this partial overlap, beta-diversity between groups was statistically significant, with 58% of the variation in microbial community structure explained by group differences (PERMANOVA-F: 3.648, R-squared: 0.578, *p* = 0.041) (Fig. 4A). Pairwise comparisons revealed a significant difference in beta-diversity between the Kisumu strain and mosquitoes that survived exposure to insecticide concentrations (1X and 10X) (PERMANOVA-F: 8.06; R-squared = 0.53; *p* = 0.02; Fig. 4B), with 53% of the variation in microbial community structure explained by the resistance status.

In *An. funestus* s.s., two distinct clusters corresponding to the FUMOZ-R and FUMOZ-HR strains were observed in the PCoA plot, although the difference in beta-diversity was not statistically significant (PERMANOVA-F: 7.31; R-squared = 0.64; *p* = 0.1; Fig. 4C). The R-squared value suggests that 64% of the variation in microbial community structure is attributed to the group differences, but this did not reach statistical significance.

### Differential abundance analysis

To pinpoint potential bacterial genera associated with resistance phenotype, a differential abundance analysis was performed using the DESeq2 test, which leverages a negative binomial distribution. Among *An. gambiae* s.s. field populations, we identified two consistent bacterial strains differentially more abundant in *Survivors_1X* mosquitoes: *Pseudomonas_1* and *Bosea*, compared to *Unexposed* and *Kisumu* groups (Additional Figs. 3 and 4; Table 2). In contrast, the genus *Serratia_2* was significantly enriched in the laboratory susceptible strain *Kisumu*, indicating their possible association with permethrin susceptibility.

**Fig. 5:**
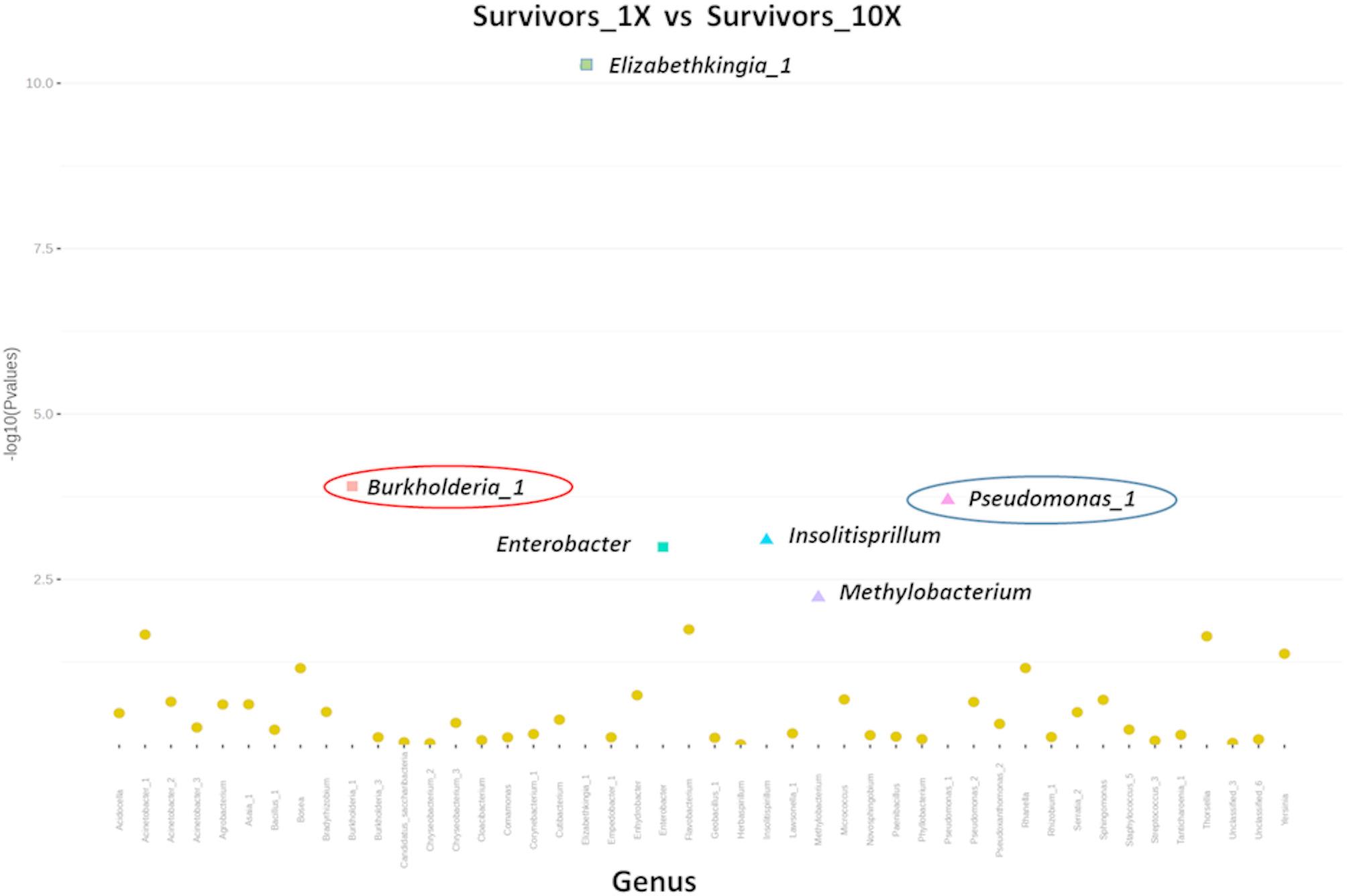
Categorical expression plot of DESeq2 results showing the differential abundance of OTUs at the genus level, based on the Log 10 transformation of *p*-value and Log2 fold change. The size and shape of the points reflect group association. Coloured squares indicate bacterial genera that are significantly associated with *Survivors_10X*. Coloured triangles represent bacterial genera that are significantly associated with *Survivors_1X*. Circles dots depict bacterial genera with no significant differential abundance between the two groups

To identify bacterial taxa potentially associated with the exacerbation of pyrethroid resistance, we analysed variations in bacterial abundance across three distinct experimental comparisons: *Survivors_10X* vs. *Unexposed*, *Survivors_10X* vs. *Kisumu*, and *Survivors_10X* vs. *Survivors_1X*. The genus *Burkholderia_1* was consistently enriched in the *Survivors_10X* group, whereas *Serratia_2* was more abundant in the *Kisumu* and *Unexposed* groups (Additional Figs. 4 and 5; Table 2). In the third comparison, the abundance of the genus *Burkholderia_1* remained elevated in the highly resistant group (*Survivors_10X)*, while *Pseudomonas_1*, *Insolitispirillum*, and *Methylobacterium* were significantly more abundant in the moderately resistant group (*Survivors_1X*). Overall, these results suggest that *Burkholderia_1* may serve as a microbial marker of high-level permethrin resistance in field populations, while *Pseudomonas_1* may be indicative of moderate resistance levels (Fig. 5; Table 2). These findings support the hypothesis that certain bacterial taxa may play a role in contributing to or modulating insecticide resistance in field-collected mosquitoes.

**Fig. 6:**
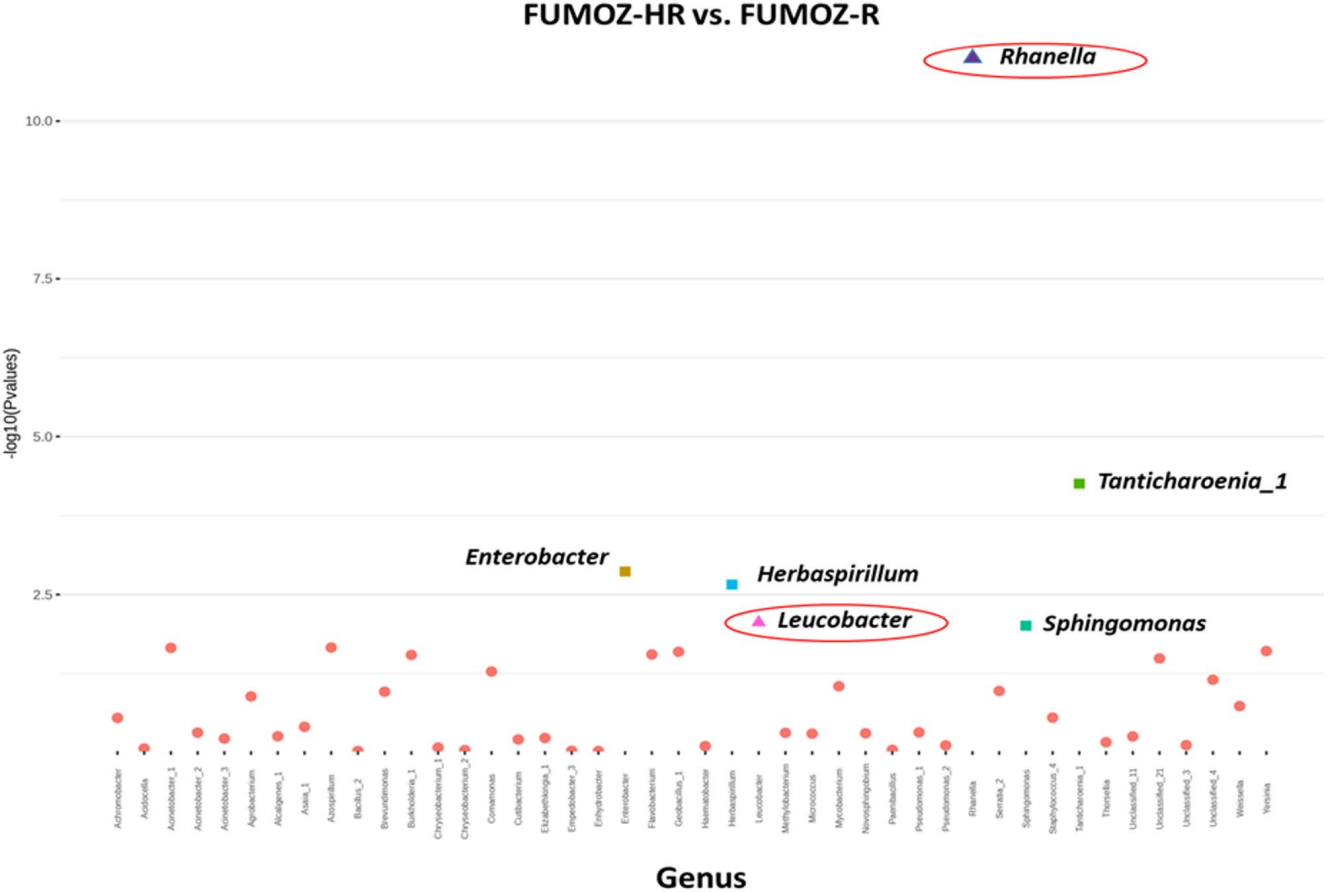
Categorical expression plot of DESeq2 analysis illustrating the differential abundance of OTUs at the genus level according to the log 10 transformation of *p*-value and Log2 fold change for the pairwise comparison FUMOZ-HR vs. FUMOZ-R. Coloured squares indicate bacterial genera that are significantly associated with FUMOZ-R. Coloured triangles represent bacterial genera that are significantly associated with FUMOZ-HR

DESeq2 analysis in *An. funestus* s.s. revealed a significant difference in bacterial abundance of between FUMOZ-HR and FUMOZ-R groups. In the FUMOZ-HR group, *Rahnella* and *Leucobacter* genera were abundant, while the relative abundance of *Enterobacter* and *Tanticharoenia_1* was very high in FUMOZ-R (Fig. 6; Table 2). These results indicate distinct bacterial abundance profiles associated with the resistance phenotypes, with specific genera potentially linked to the escalation of pyrethroid resistance.

**Table 2:** DESeq2-based differential abundance analysis revealed the most significantly enriched bacterial taxa

Bacterial taxa	DESeq2 results			Bacterial abundance		
*Survivors_1X* vs. *Unexposed*						
Genera	*p*-value	Log2FC	LfcSE	*Survivors_1X*	*Unexposed*	Total
***Pseudomonas_1***	**0.01**	**−3.89**	**1.73**	**121,900**	**7251**	**129,151**
* Bosea*	0.02	−2.17	0.94	5345.2	848.33	6193.53
* Aeromonas_2*	3.00E-09	9.97	1.68	0	5021.9	5021.9
* Elizabethkingia_1*	4.40E-07	10.23	2.02	1603	1,522,800	1,524,403
* Serratia_2*	2.30E-05	5.64	1.33	3326.3	138,080	141406.3
*Survivors_1X* vs. *Kisumu*						
Genera	*p*-value	Log2FC	LfcSE	*Survivors_1X*	Kisumu	Total
* Acidocella*	5.20E-08	−9.82	1.8	536,950	240.12	537190.12
***Burkholderia_1***	**1.01E-06**	**−9.7**	**1.98**	**17,140**	**0**	**17,140**
* Tanticharoenia_1*	1.10E-07	−6.86	1.29	719,180	6038.4	725218.4
* Bosea*	0.0002	−8.04	2.02	5346.8	0	5346.8
* Asaia_1*	0.0007	−4.09	1.21	7,980,000	563,980	8,543,980
***Pseudomonas_1***	**0.002**	**−4.27**	**1.4**	**121,940**	**6371.6**	**128311.6**
* Elizabethkingia_1*	3.50E-17	12.54	1.48	1603.3	5,639,600	5641203.3
* Burkholderia_2*	6.40E-12	13.95	2.03	0	111,640	111,640
* Thorsellia*	1.40E-08	13.26	2.33	11.06	181,620	181631.06
* Aquabacterium*	2.70E-07	11.07	2.15	0	16,357	16,357
* Serratia_2*	4.20E-07	7.93	1.56	3326.7	1,151,900	1155226.7
*Survivors_10X* vs. *Unexposed*						
Genera	*p*-value	Log2FC	LfcSE	*Survivors_10X*	*Unexposed*	Total
* Unclassified_1*	1.10E-27	−29.63	2.71	151,120	0	151,120
*** Burkholderia_1***	**0.0006**	**−5.67**	**1.67**	**128,850**	**10,672**	**139,522**
* Chryseobacterium_1*	4.22E-19	25.93	2.9	0	136,530	136,530
*Survivors_10X* vs. *Kisumu*						
Genera	*p*-value	Log2FC	LfcSE	*Survivors_10X*	Kisumu	Total
* Unclassified_1*	3.20E-25	−28.61	2.75	151,120	0	151,120
***Burkholderia_1***	**3.80E-10**	**−14.47**	**2.31**	**128,850**	**0**	**128,850**
* Acidocella*	1.20E-07	−11.17	2.11	305,750	240.08	305990.08
* Tanticharoenia_1*	0.00001	−6.51	1.48	160,920	6035.9	166955.9
* Asaia_1*	0.001	−4.93	1.9	5,317,400	563,850	5,881,250
* Stenotrophomonas_2*	1.60E-09	14.58	2.41	0	365,090	365,090
* Burkholderia_2*	3.60E-08	12.16	2.2	0	111,590	111,590
* Aeromonas_1*	1.10E-06	11.63	2.38	0	49,996	49,996
* Serratia_2*	4.9 E-05	7.08	1.74	3769.7	1,151,700	1155469.7
*Survivors_1X* vs. *Survivors_10X*						
Genera	*p*-value	Log2FC	LfcSE	*Survivors_10X*	*Survivors_1X*	Total
*** Pseudomonas_1***	**0.0001**	**−4.42**	**1.18**	**2436.8**	**121,950**	**124386.8**
* Elizabethkingia_1*	6.31E-11	10.84	1.65	2,383,200	1603.8	2384803.8
***Burkholderia_1***	**0.0001**	**4.95**	**1.31**	**133,950**	**17,143**	**151,093**
* Enterobacter*	0.001	4.01	1.24	1,498,300	277,650	1,775,950
FUMOZ-HR vs. FUMOZ-R						
Genera	*p*-value	Log2FC	LfcSE	FUMOZ-HR	FUMOZ-R	Total
***Rahnella***	**9.73E-12**	**−15.954**	**2.34**	**4,835,500**	**290.7**	**4835790.7**
***Leucobacter***	**0.008**	**−7.6**	**2.91**	**62,384**	**1773.8**	**64157.8**
* Tanticharoenia_1*	7.79E-05	10.02	2.47	0	33,374	33,374
* Enterobacter*	0.001	3.22	1.04	79,466	1,633,600	1,713,066
* Sphingomonas*	0.009	2.87	1.1	7614.6	133,320	140934.6

Bacteria in bold are potential microbial markers associated with pyrethroid resistance

### Efficacy of the antibiotic treatment and survival assay

**Fig. 7:**
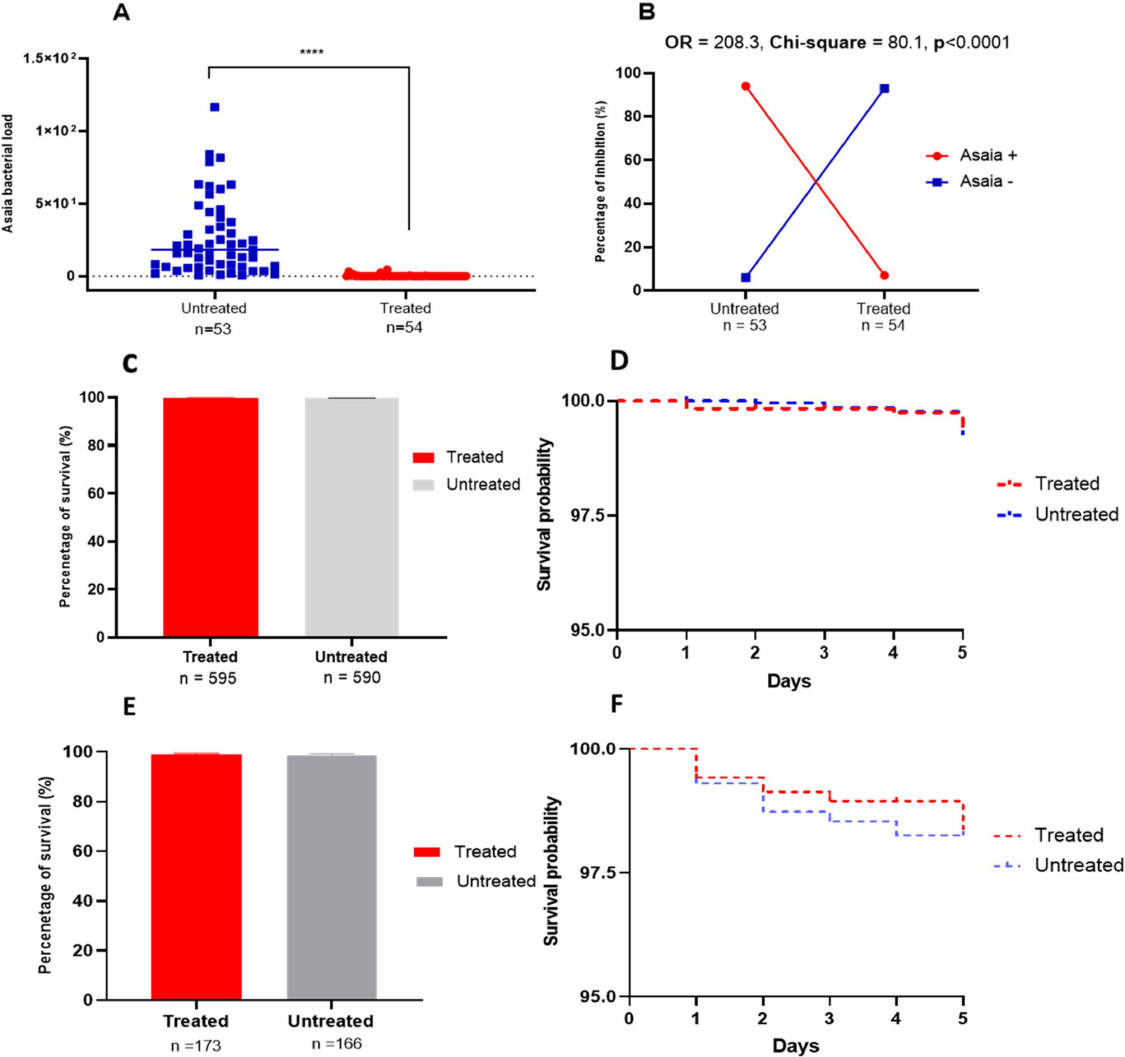
Bacterial quantification and survival assessment in *Anopheles* mosquitoes: **A** Relative abundance of *Asaia* symbiont after antibiotic treatment of *Anopheles* mosquitoes. **B** Association between the antibiotic treatment and the bacterial inhibition. **C** Cumulative survival rate of Fumoz-R strain after 5 days of antibiotic treatment. **D** Daily survival curve of Fumoz-R after antibiotic treatment. **E** Cumulative survival rate of *An. gambiae* field mosquitoes during 5 days of antibiotic treatment. **F** Daily survival curve of *An. gambiae* field mosquitoes after antibiotic treatment. The calculated odds ratios (OR) reinforce the statistical significance of these findings, with *p*-values below 0.05 highlighting their robustness. ****: Significance at *p*-value < 0.0001

To verify the effectiveness of the antibiotic treatment, we assessed the bacterial load of *Asaia* spp. We observed a drastic reduction in its abundance in the treated group compared to the untreated counterpart, as demonstrated by the Mann-Whitney U test (U = 25, *p* < 0.0001; Fig. 7A). Furthermore, there was a substantial decline in the proportion of individuals infected with *Asaia* spp. in the treated group, highlighting the treatment’s efficacy (OR = 208.3; Chi-square = 80.1; *p* < 0.0001; Fig. 7B). To evaluate the effect of the antibiotic treatment on the survival of *Anopheles* mosquitoes, we monitored the longevity of 768 mosquitoes from the treated group (*An. gambiae* s.s. = 595. *An. funestus* s.s. = 173) and 756 mosquitoes from the untreated group (*An. gambiae* s.s. = 590. *An. funestus* s.s. = 166) over 5 days. The antibiotic treatment did not significantly impact survival rates for either species, as evidenced by results for *An. gambiae* s.s. ([Log Rank test]: Chi-square = 0.06; *p* = 0.79) and *An. funestus* s.s. ([Log Rank test]: Chi-square = 0.4; *p* = 0.51) (Fig. 7C-F).

### Cytochrome P450 expression levels after the antibiotic treatment

**Fig. 8:**
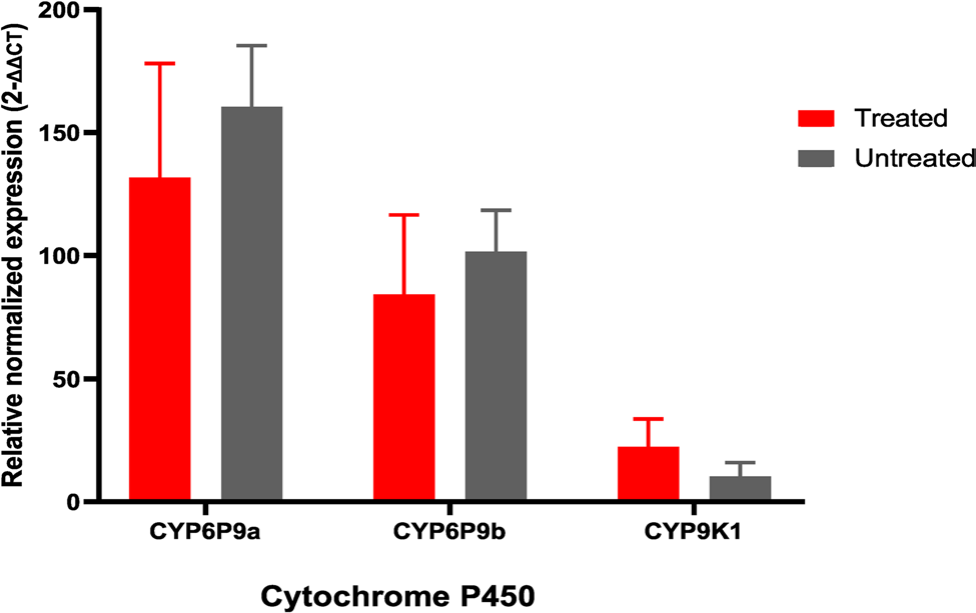
Impact of antibiotic treatment on the expression of key detoxification genes (*CYP6P9a*, *CYP6P9b* and *CYP9K1*) in *Anopheles* mosquitoes. Changes in gene expression were quantified, with error bars representing the standard errors of the mean fold change (SEM) and a 95% confidence interval (CI), *p*-value < 0.05

To investigate whether antibiotic treatment attenuates the expression of detoxification-related genes, we analysed the expression levels of cytochrome P450s, key mediators of pyrethroid resistance, between treated and untreated groups. In the FUMOZ-R strain **(***An. funestus* s.s.), the *CYP6P9a* gene exhibited an average fold change of 131.5 ± 46.6 in antibiotic-treated individuals, compared to 160.4 ± 24.96 in the untreated individuals, however, this difference was not statistically significant (*p* = 0.36; Welch’s t-test). A similar pattern was observed for the *CYP6P9b* gene, which showed fold changes of 84 ± 32.45 and 101.4 ± 16.96 for treated and untreated mosquitoes, respectively, again without statistical significance (*p* = 0.41; Welch’s t-test). In contrast, the expression of *CYP9K1* gene was elevated in the treated group (22.25 ± 11.49) compared to the untreated group (10.11 ± 5.77), however this difference was not statistically significant (*p* = 0.11) (Fig. 8).

### Susceptibility profile after antibiotic treatment

**Fig. 9:**
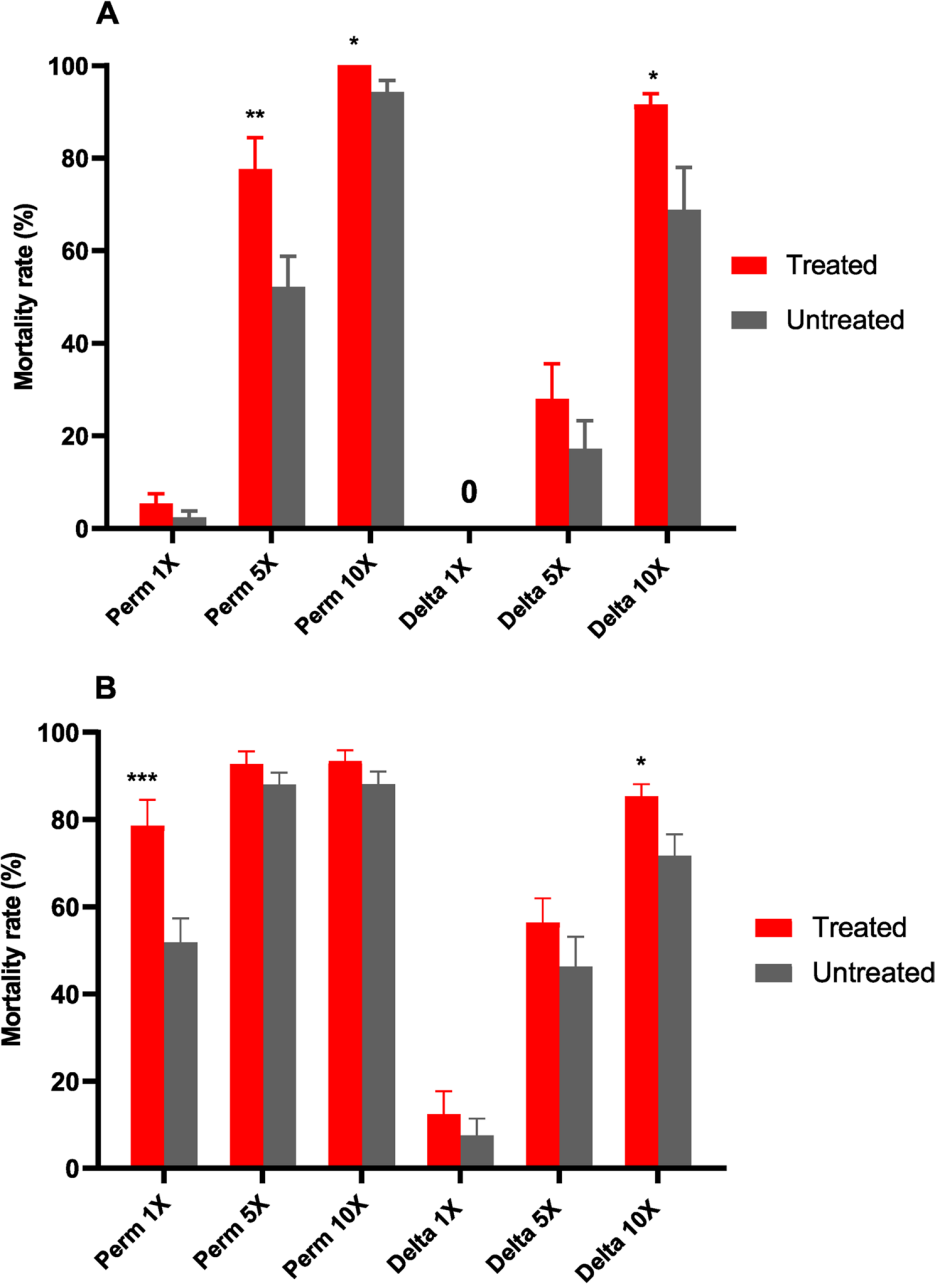
Impact of antibiotic treatment on the resistance profile in *Anopheles* mosquitoes: Susceptibility profiles of (**A**) Nkolondom *An. gambiae* s.s. field mosquitoes and (**B**) FUMOZ-R strain to pyrethroids after antibiotic treatment. Histograms show mean mortality rates post-exposure to insecticides, with error bars indicating standard errors of the mean mortality rate (SEM) and a 95% confidence interval (CI). Statistical analysis revealed significant differences, with *p*-values < 0.05, < 0.01, and < 0.001 denoted by *,**,***. The bioassay included at least three replicates, each with over 20 mosquitoes. Perm: Permethrin, Delta: Deltamethrin

The impact of antibiotic treatment on insecticide susceptibility was evaluated, revealing a marked increase in mortality rates among treated mosquitoes exposed to both permethrin and deltamethrin at all tested concentrations, compared to untreated counterparts. In the *An. gambiae* s.s., mortality rates significantly differed among the two groups exposed to permethrin at 5X (Student-t test: t (*df*) = 2.69; *p* = 0.008), 10X (Student-t test: t (*df*) = 2.8; *p* = 0.03) and deltamethrin 10X (Student-t test: t (*df*) = 2.49; *p* = 0.01), as illustrated in Fig. 9A. Similarly, *An. funestus* s.s. exhibited heightened susceptibility, with significant increases in mortality rates recorded for permethrin at 1X (Student-t test: t (*df*) = 3.29; *p* = 0.001), and deltamethrin 10X (Student-t test: t (*df*) = 2.44; *p* = 0.01 shown in Fig. 9B.

## Discussion

Elucidating the role of microbiome in insecticide resistance is an important step in understanding the evolutionary and biological processes that enable mosquitoes to evade insecticide-based control interventions. By exploring the intricate interactions between mosquitoes and their symbiotic microorganism, we can gain valuable insights that may inform the design of innovative strategies to manage highly resistant mosquito populations. The present study investigated how the *Anopheles* microbiome impacts mosquitoes’ survival to pyrethroids using both field populations and laboratory strains of two major African vectors, *An. gambiae* s.s. and *An. funestus* s.s.

### Elevated resistance intensity to pyrethroid in both *Anopheles* species

A stepwise evaluation of pyrethroid resistance in field-collected *An. gambiae* s.s. mosquitoes from Nkolondom revealed a high intensity of resistance to permethrin and deltamethrin. The *An. funestus* FUMOZ-HR laboratory strain was already selected against high doses of insecticides, and the within-resistance intensity was confirmed. These field findings are consistent with previous reports of worsening pyrethroid resistance in African *Anopheles* populations, particularly within Cameroon [13, 14, 52]. The indiscriminate use of insecticides in agricultural and public health contexts remains a critical factor driving the selection pressure for resistance in these malaria vectors [13, 14]. The genetic mechanisms underlying this high level of pyrethroid resistance are mixed, confirming the fact that insecticide resistance is polygenic. In *An. gambiae* s.s., one prominent genetic adaptation is resistance through target site modifications, such as *kdr L995F* (*L995F/L995F*) and *kdr N1570Y* loci (formerly *N1575Y*), and these were found in the Nkolondom locality [13, 53]. Additionally, enzymatic detoxification can reinforce resistance, mediated by mono-oxygenases, cytochromes P450, such as *CYP6P3* in *An. gambiae* s.s [54–56]. *CYP6P9a/b* [48, 57–59] and *CYP9K1* in *An. funestus* s.s [60–62]. Elevated glutathione-S-transferases (GSTs) expression has also been implicated in insecticide resistance of both vectors, *An. funestus* s.s. and *An. gambiae* s.s [63–65]. Recently, agnostic approaches have provided new insights and evidence of the role of mosquito microbiome in pyrethroid resistance, but we investigated further if they contribute to resistance escalation.

### *Anopheles* mosquitoes presented a core microbiota and similar bacterial diversity between resistance phenotypes

A total of one hundred and eighty OTUs, spanning 6 phyla, 33 families and 59 genera were identified across the mosquito samples tested, providing a valuable update on the *Anopheles* microbiota composition. The predominant bacterial phyla identified were Gram-negative *Proteobacteria* and *Firmicutes*. Among the bacterial families, *Acetobacteraceae*, *Weeksellaceae*, and *Enterobacteriaceae* stood out with the genera *Asaia*,* Elizabethkingia*, *Enterobacter* and *Rahnella* being particularly prevalent. These findings align with previously published data from African *Anopheles* mosquitoes [23, 66–68]. These common bacteria are known as the “core microbiota” in field and laboratory adult mosquitoes [23, 66–68]. Their dominance is likely due to their critical role in mosquito life traits including fecundity, reproduction, vectorial competence and immunity [69]. These microbial associations underline the intricate relationships between mosquitoes and their microbiota, revealing the complexity in elucidating certain phenotypes exhibited by mosquitoes with potential impact on vector control strategies.

The comparison of bacterial richness and evenness (alpha diversity) between phenotypes of *Anopheles* species showed no significant differences. This aligns with findings from a Kenyan study indicating similar microbiota in resistant and susceptible *An. gambiae* mosquitoes [70]. Similar patterns were also observed in *Aedes aegypti*, where resistance phenotypes exhibited minimal differences in bacterial community structure [71]. The homogeneity in alpha diversity may stem from comparable breeding conditions and physiological traits, as mosquitoes from shared aquatic habitats tend to have similar microbiota [24]. Additionally, the pooling of mosquito samples during sequencing may have constrained our ability to detect finer-scale variations in microbial communities, such as individual-level differences in bacterial abundance or rare taxa potentially associated with resistance phenotypes [72]. However, resistant mosquitoes exhibited a lower level of alpha diversity, possibly as a result of insecticide selection pressure that may favour bacteria capable of metabolising insecticides [21, 73]. Differences in microbial composition were observed between the susceptible Kisumu strain and the permethrin-resistant *An. gambiae* s.s. field mosquitoes, reflecting genetic and environmental influences on microbiota. Future research should focus on long-term studies that monitor microbial shifts before, during, and after insecticide exposure, along with the inclusion of controlled environments to more effectively isolate the role of microbial communities in resistance mechanisms.

### Bacterial taxa associated with pyrethroid-resistant *Anopheles* populations

Differential abundance tests identified several bacterial genera associated with insecticide resistance, notably *Rahnella* and *Leucobacter* genera, which correlate with escalated levels of deltamethrin resistance in *An. funestus* s.s. This finding aligns with Chen et al. (2024), who reported an increased abundance of *Rahnella*, alongside *Azospirillum*, *Cedecea*, and *Agromyces* in a highly resistant lambda-cyhalothrin-selected FUMOZ strain [67]. *Rahnella* has been shown to degrade clothianidin (a neonicotinoid) at various concentrations, mitigating its toxic effects on honeybee colonies [74]. Moreover, specific strains of this bacteria can completely degrade the organophosphate profenofos into metabolites such as 4-bromo-2-chlorophenol, phosphoric acid, and 3,4-dimethylbenzoic acid, establishing it as an effective bioremediant agent [75]. In the present study, the deltamethrin selection pressure applied to the FUMOZ strain appears to promote *Rahnella* proliferation over other taxa, suggesting its functional role in mitigating insecticide toxicity. This could appear through enzymatic breakdown [76], biotransformation or bioaccumulation process [77]. Future genomic and transcriptomic research should explore the mechanisms by which *Rahnella*, contributes to pyrethroid resistance. In *An. gambiae* s.s., a high abundance of *Pseudomonas_1* was found in permethrin-resistant mosquitoes, correlating with enhanced survival post-exposure to insecticides. *Pseudomonas* species are known for their hydrolase activity (Esterases), enabling them to degrade various insecticides in their hosts and utilise them as sources of carbon, phosphorus, or nitrogen [78–80]. For instance, a strain of *P. aeruginosa* isolated from the cockroach *Blatta orientalis* was capable of degrading 88.5% of endosulfan (an organochlorine insecticide) in vitro within 10 days [81]. Additionally, studies suggest that *Pseudomonas* may enhance the mosquito’s detoxification cascade by promoting the expression of key enzymes [82]. Interestingly, our findings revealed a strong association between *Burkholderia* abundance and permethrin resistance intensity. A key study by Kikuchi et al. (2012) introduced the concept of ‘symbiont-mediated insecticide resistance’, demonstrating that *Burkholderia* bacteria can rapidly colonise their hosts (stink bug, *Riptortus pedestris*), breakdown fenitrothion and confer resistance to this class of insecticide [28]. Given *Burkholderia*’s ability to efficiently degrade organophosphate, it’s plausible that certain strains may also metabolise other insecticide classes, such as pyrethroids, enabling mosquitoes to withstand higher concentrations of these chemicals. This assumption is supported by studies demonstrating that specific bacterial genera, notably *Acinetobacter*, possess metabolic versatility allowing the degradation of a broad spectrum of xenobiotic substances, including malathion (an organophosphate) [83] and deltamethrin (a pyrethroid) [84]. These findings lay the groundwork for further investigations into the roles of *Pseudomonas* and *Burkholderia* in pyrethroid degradation, potentially shedding light on their contribution to the exacerbation of insecticide resistance and the development of possible cross-resistance to other insecticide classes in *Anopheles* populations.

### Partial restoration of pyrethroid susceptibility in the resistant mosquitoes supports the role of bacterial microbiota in resistance escalation

The antibiotic treatment significantly reduced *Asaia* spp. in the treated group. Similar decreases in bacterial load have been reported in *An. darlingi* and *Aedes albopictus* [85, 86]. While these results suggest that antibiotic treatment can alter microbial abundance, a key limitation of this study is the absence of 16 S rRNA sequencing, which would have enabled a comprehensive assessment of the full extent of bacterial depletion and potential shifts in overall microbiota composition.

Following treatment, our results revealed that the FUMOZ-R strain exhibited increased susceptibility to permethrin at the diagnostic dose (1X), correlated with the low expression level of the main metabolic resistance genes *CYP6P9a* and *CYP6P9b* in the treated group. This suggests that pyrethroid resistance may be influenced by the mosquitoes’ microbiome, as bacteria can enhance metabolic detoxification and modulate the immune response to mitigate oxidative stress from insecticide exposure [86, 87]. To further substantiate this hypothesis, future studies should consider introducing targeted bacteria species to assess their direct impact on cytochrome P450 enzyme activity. Additionally, our results indicated that antibiotic treatment had minimal impact at higher diagnostic doses of permethrin, suggesting that the core biochemical resistance mechanisms remain constant even at elevated insecticide doses. In contrast to permethrin, a more pronounced effect of antibiotic treatment was observed at higher concentrations of deltamethrin, indicating that its efficacy in increasing susceptibility may be insecticide-specific. This discrepancy could be attributed to the structural difference between Type I (permethrin) and Type II (deltamethrin) pyrethroids, which are known to engage distinct detoxification pathways. Consequently, microbiome-mediated modulation of resistance may differentially affect mosquito survival depending on the chemical class of insecticide.

In *An. gambiae* s.s., antibiotic treatment increased susceptibility to both insecticides at higher concentrations, corroborating previous studies conducted on *An. stephensi*,* An. arabiensis* and *Ae. aegypti* [30, 31, 88]. This implies that reducing the bacterial community in *An. gambiae* s.s. could restore susceptibility to pyrethroids in the field. This observation raises new questions about the molecular dynamics of insecticide resistance and further supports the role of internal bacteria in exacerbating pyrethroid resistance. It highlights the need for further research to explore how microbial communities influence resistance to current and new insecticides and how their manipulation could enhance vector control strategies, including the design of new formulations that incorporate bacterial-neutralising agents to mitigate microbiota-driven resistance.

## Conclusion

The contribution of microbiota to the widespread escalation of pyrethroid resistance in malaria vectors in Africa has remained unclear. This study reported the enrichment of specific bacterial taxa associated with the aggravation of pyrethroid resistance (*Rahnella*, *Leucobacter*,* Pseudomonas_1*, and *Burkholderia_1*). These bacteria may play a complex host-specific role in determining the resistance phenotype involving several different and simultaneous pathways, such as direct degradation of the insecticide, changes in the host immune system, alterations in the midgut, and increased expression of genes that detoxify pyrethroids. The present work advances our understanding of the evolution of insecticide resistance in malaria vectors, particularly their recently acquired ability to withstand higher doses of insecticides, which could help manage resistance against new and future insecticides.

## Supplementary Information

Additional file 1.

## Data Availability

Sequence data of the targeted bacteria were deposited in GenBank of NCBI database under accession numbers PV014963-PV014967. All analytical code used in this study is publicly available in the corresponding GitHub repository: https://github.com/Djondji/Metagenomics-16S.
