# Supplementary material for: Evidence of microbiome contribution to the escalation of pyrethroid resistance in the major malaria vectors Anopheles gambiae s.s. and Anopheles funestus s.s
Source: BMC Microbiol. 2025 Jul 2;25:384. doi: 10.1186/s12866-025-04114-0 (PMC12220112; doi:10.1186/s12866-025-04114-0)
Supplement: Supplementary file 1 — Additional file 1. [file 12866_2025_4114_MOESM1_ESM.docx]

**Additional Tables**

**Additional Table S. 1.** Number of samples used for the 16S rRNA sequencing

| Species | Phenotypes | Pools | Number of mosquitoes | Number of replicates |
| --- | --- | --- | --- | --- |
| *An. gambiae* s.s. | ***Unexposed*** | Pool_1 | 9 | 3 |
|  |  | Pool_2 | 9 |  |
|  |  | Pool_3 | 9 |  |
|  | ***Survivors_1X*** | Pool_4 | 9 | 3 |
|  |  | Pool_5 | 9 |  |
|  |  | Pool_6 | 9 |  |
|  | ***Survivors_10X*** | Pool_7 | 9 | 3 |
|  |  | Pool_8 | 9 |  |
|  |  | Pool_9 | 9 |  |
|  | ***Susceptible Kisumu*** | Pool_16 | 9 | 3 |
|  |  | Pool_17 | 9 |  |
|  |  | Pool_18 | 9 |  |
| *An. funestus* s.s. | ***FUMOZ-R*** | Pool_10 | 9 | 3 |
|  |  | Pool_11 | 9 |  |
|  |  | Pool_12 | 9 |  |
|  | ***FUMOZ-HR*** | Pool_13 | 9 | 3 |
|  |  | Pool_14 | 9 |  |
|  |  | Pool_15 | 9 |  |
| Blank | **Ethanol 70%** | Pool_19 | 3 | 1 |
|  | **PBS solution** | Pool_20 | 3 | 1 |
|  | **Negative control** | Pool_21 | 3 | 1 |
| Total |  |  | 162 mosquitoes, 3 blanks | 21 pools |

**Additional Table S. 2. List of primers**

|  | Oligonucleotides names | Sequences (5'-3') |
| --- | --- | --- |
| *CYP6P9a* (qPCR) | *CYP6P9a* qPCR_F | CAGCGCGTACACCAGATTGTGTAA |
|  | *CYP6P9a* qPCR_R | TCACAATTTTTCCACCTTCAAGTAATTACCCGC |
| *CYP6P9b* (qPCR) | *CYP6P9b* qPCR_F | CAGCGCGTACACCAGATTGTGTAA |
|  | *CYP6P9b* qPCR_R | TTACACCTTTTCTACCTTCAAGTAATTACCCGC |
| *CYP9K1* (qPCR) | *CYP9K1*qPCR_F | CCGACACGTGGTGATGGATAC |
|  | *CYP9K1*qPCR_R | CGTCGTCGGTCCAGTCAAC |
| *Asaia* 16S RNA (qPCR) | AsaH1_F | AAGGGCGCGTAGGCGGTTTA |
|  | Asar_R | CCACCGGTGTTCTTCCCAAT |
| Housekeeping genes | Rsp7_F (*An. gambiae* s.s.) | CCACCATCGAACACACAAAGTTGA |
|  | Rsp7_R (*An. gambiae* s.s.) | TGCTGCAAACTTCGGCTATTC |
|  | EFqPCR_F (*An. gambiae* s.s.) | GGCAAGAGGCATAACGATCAATGCG |
|  | EFqPCR_R (*An. gambiae* s.s.) | GTCCATCTGCGACGCTCCGG |
|  | Rsp7_F (*An. funestus* s.s.) | GTGTTCGGTTCCAAGGTGAT |
|  | Rsp7_R (*An. funestus* s.s.) | TCCGAGTTCATTTCCAGCTC |
|  | Actin_F (*An. funestus* s.s.) | TTAAACCCAAAAGCCAATCG |
|  | Actin_R (*An. funestus* s.s.) | ACCGGATGCATACAGTGACA |
| *F= Forward primer, R= Reverse primer, Rsp = Ribosomal protein, EF= Elongation factor, CYP= Cytochrome | | |

**Additional Table S. 3. Percentage of sequencing coverage in overall samples**

| Phenotypes | Pools | Number of singletons | Reads counts | Sequencing coverage |
| --- | --- | --- | --- | --- |
| *Unexposed* | Pool_1 | 1 | 202107 | 99.9995 |
|  | Pool_2 | 4 | 133312 | 99.9969 |
|  | Pool_3 | 2 | 170052 | 99.9988 |
| *Survivors_1X* | Pool_4 | 2 | 301514 | 99.99933 |
|  | Pool_5 | 0 | 429697 | 100 |
|  | Pool_6 | 1 | 222463 | 99.9995 |
| *Survivors_10X* | Pool_7 | 2 | 452715 | 99.9995 |
|  | Pool_8 | 2 | 195042 | 99.9989 |
|  | Pool_9 | 2 | 249395 | 99.9991 |
| *FUMOZ-R* | Pool_10 | 0 | 146397 | 100 |
|  | Pool_11 | 0 | 256152 | 100 |
|  | Pool_12 | 2 | 229053 | 99.99912 |
| *FUMOZ-HR* | Pool_13 | 4 | 146632 | 99.99727 |
|  | Pool_14 | 5 | 114537 | 99.99563 |
|  | Pool_15 | 9 | 96711 | 99.99069 |
| *Susceptible Kisumu* | Pool_16 | 3 | 72060 | 99.9958 |
|  | Pool_17 | 4 | 166530 | 99.9975 |
|  | Pool_18 | 0 | 65059 | 100 |

**Additional Figures**


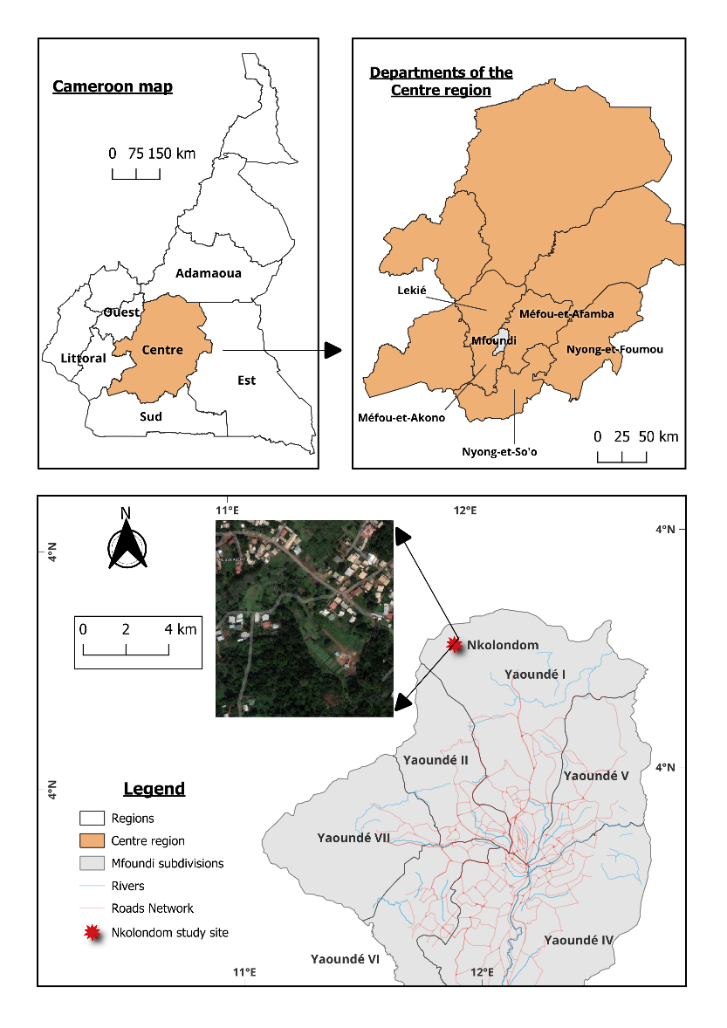


**Additional Figure 1. Illustrative map of Nkolondom agricultural setting.** This map was constructed using the software QGIS version 3.38-Lima. <https://qgis.org/>. The larvae stage of An. gambiae mosquitoes were collected in a large drain charnel.


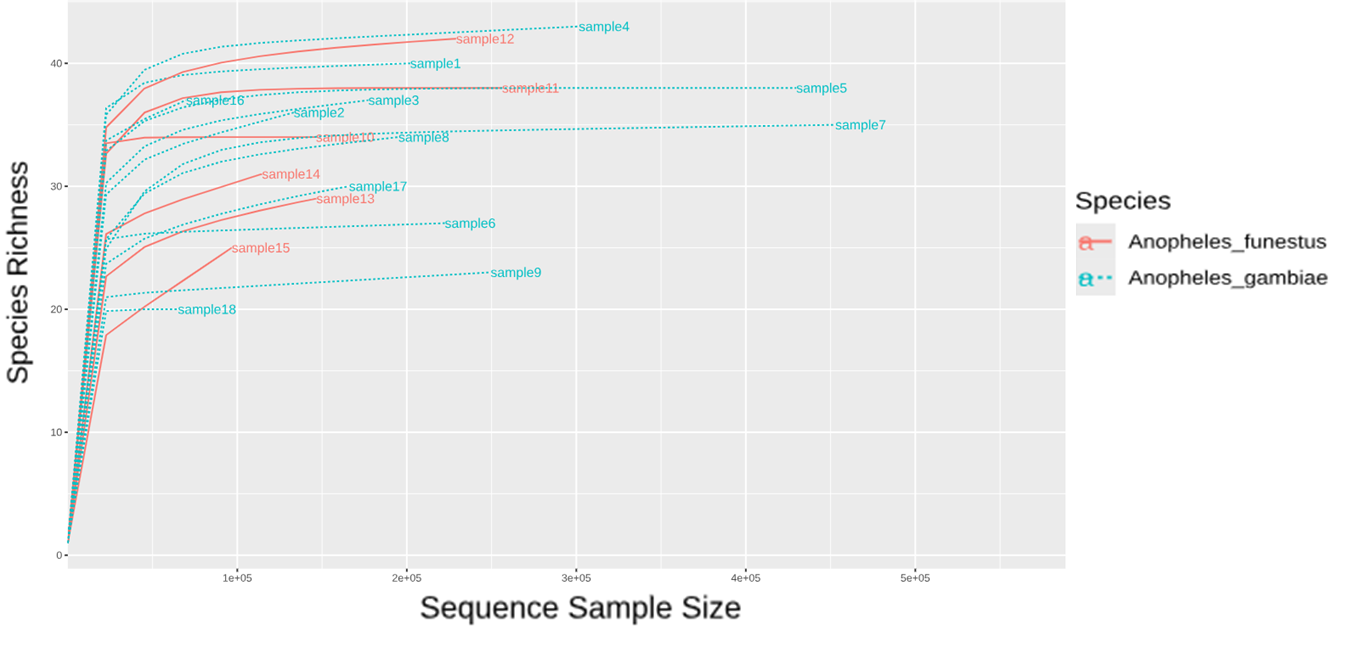


**Additional Figure 2*.*** Rarefaction curves of the libraries were obtained after sequencing according to *Anopheles* species.

**
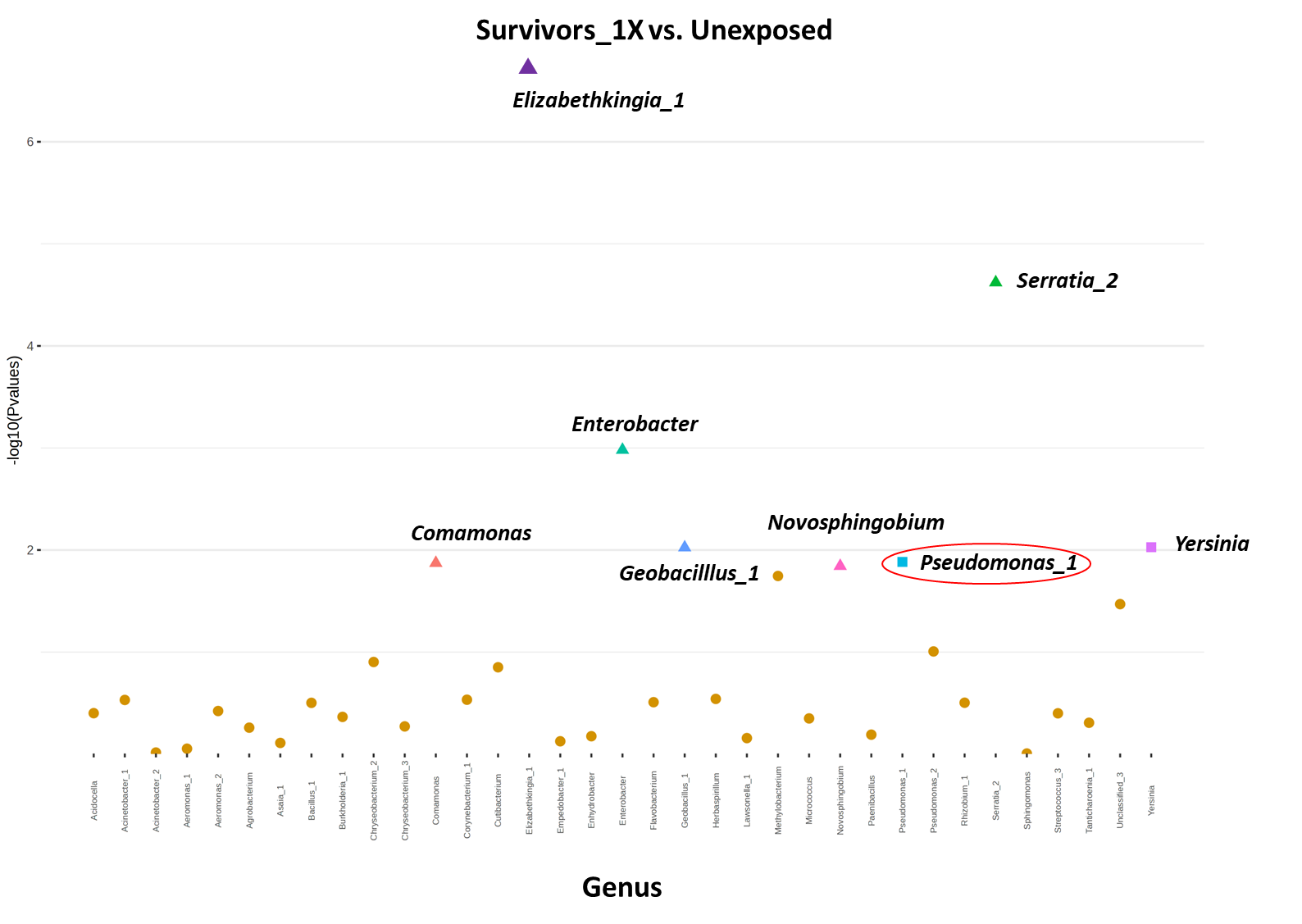
**

**Additional Figure 3*.*** Categorical expression plot of DESeq2 results showing the differential abundance of OTUs identified at the genus level according to the p-value and Log2 fold change of the *An. gambiae* s.s. mosquitoes: Pairwise comparison *Survivors_1X* vs. *Unexposed*. Coloured squares indicate bacterial genera that are significantly associated with *Survivors_1X*. Coloured triangles represent bacterial genera that are significantly associated with *Unexposed* (FDR-adjusted p-value  [< 0.05) (https://www.microbiomeanalyst.ca/MicrobiomeAnalyst/).](%20%3c%200.05)%20(https://www.microbiomeanalyst.ca/MicrobiomeAnalyst/).%20)


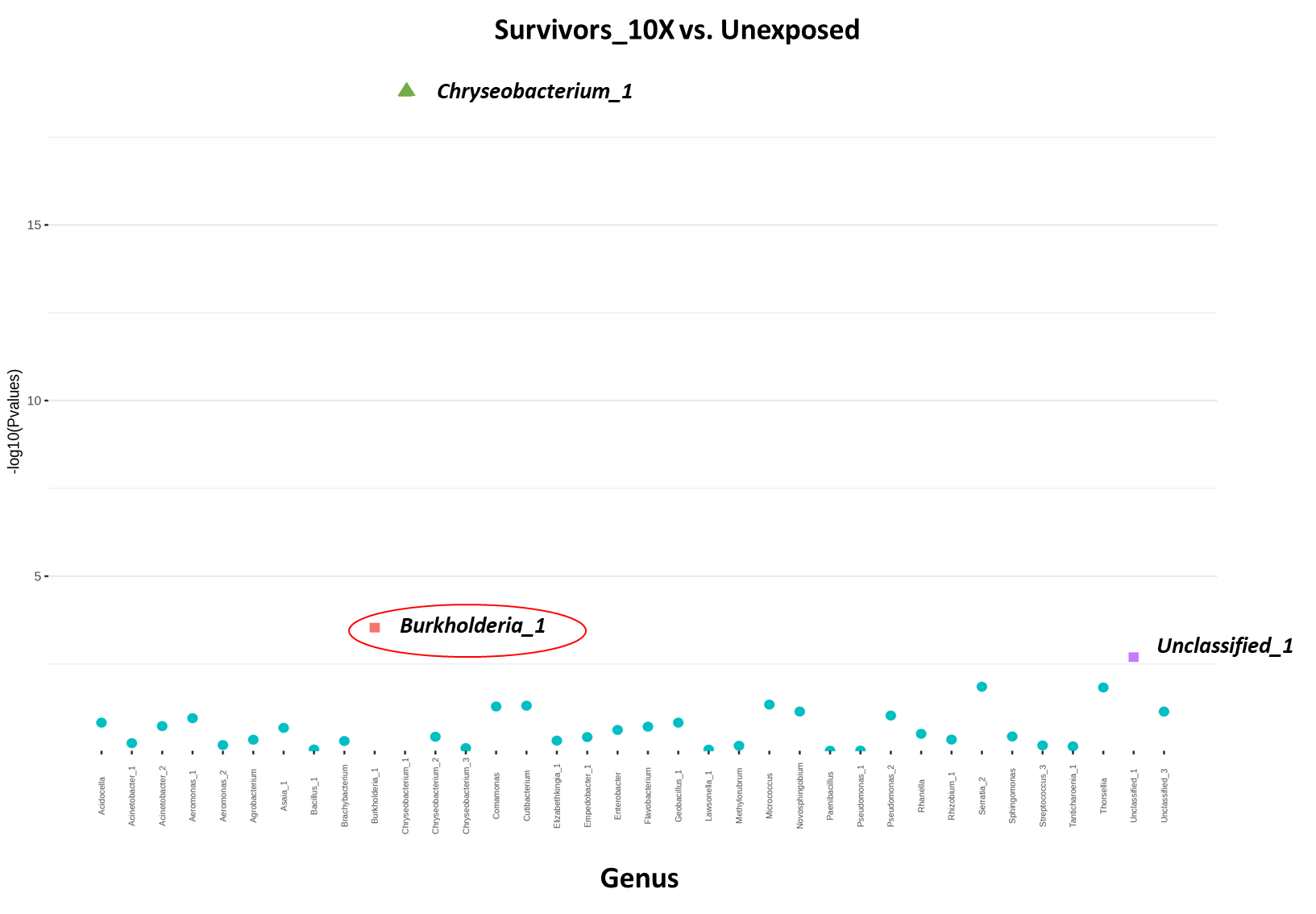


**Additional Figure 5*.*** Categorical expression plot of DESeq2 results showing the differential abundance of OTUs identified at the genus level according to the p-value and Log2 fold change of the *An. gambiae* s.s. mosquitoes: Pairwise comparison *Survivors_10X* vs. *Unexposed*. Coloured squares indicate bacterial genera that are significantly associated with *Survivors_10X*. Coloured triangles represent bacterial genera that are significantly related to *Unexposed (*FDR-adjusted p-value < 0.05) (https://www.microbiomeanalyst.ca/MicrobiomeAnalyst/).

**
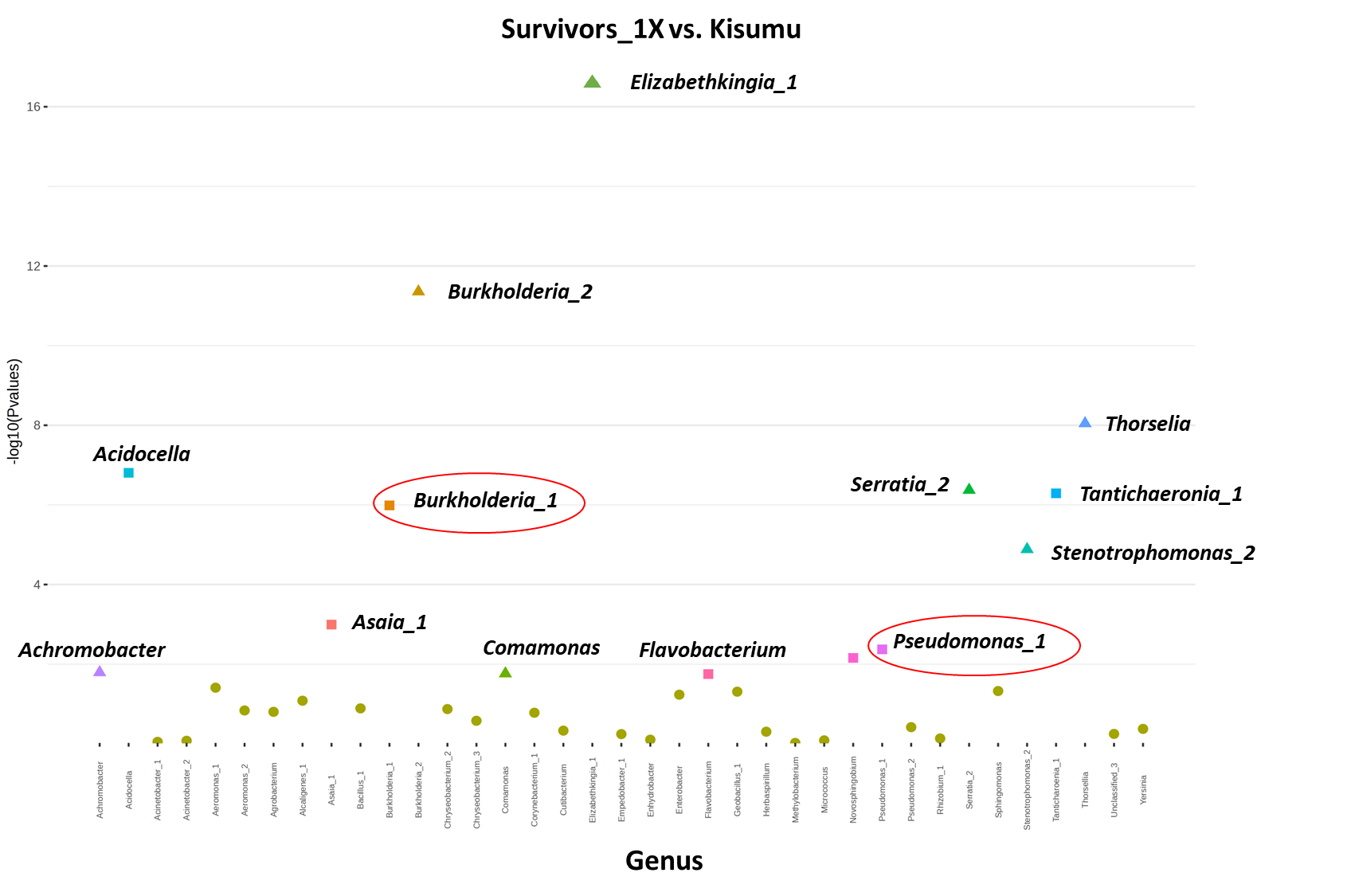
**

**Additional Figure 4*.*** Categorical expression plot of DESeq2 results showing the differential abundance of OTUs identified at the genus level according to the p-value and Log2 fold change of the *An. gambiae* s.s. mosquitoes: Pairwise comparison *Survivors_1X* vs *Kisumu*. Coloured squares indicate bacterial genera that are significantly associated with *Survivors_1X*. Coloured triangles represent bacterial genera that are significantly associated with *Kisumu (*FDR-adjusted p-value < 0.05) (https://www.microbiomeanalyst.ca/MicrobiomeAnalyst/).


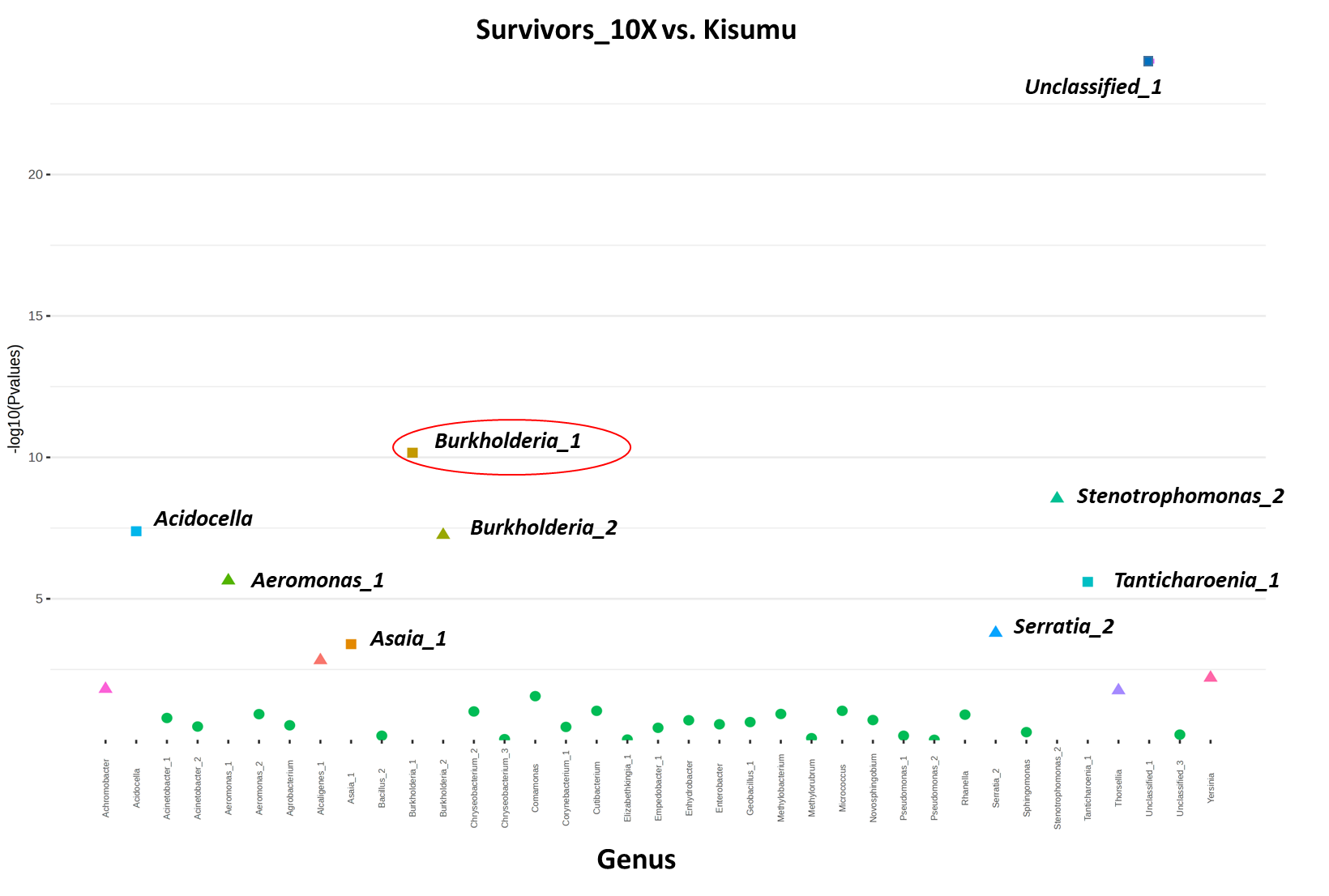


**Additional Figure 6*.*** Categorical expression plot of DESeq2 results showing the differential abundance of OTUs identified at the genus level according to the p-value and Log2 fold change of the *An. gambiae* s.s. mosquitoes: Pairwise comparison *Survivors_10X* vs. *Kisumu*. Coloured squares indicate bacterial genera that are significantly associated with *Survivors_10X*. Coloured triangles represent bacterial genera that are significantly associated with *Kisumu (*FDR-adjusted p-value  [< 0.05) (https://www.microbiomeanalyst.ca/MicrobiomeAnalyst/).](%20%3c%200.05)%20(https://www.microbiomeanalyst.ca/MicrobiomeAnalyst/).%20)
